# Early diagnosis of bladder cancer by photoacoustic imaging of tumor-targeted gold nanorods

**DOI:** 10.1016/j.pacs.2022.100400

**Published:** 2022-08-30

**Authors:** Elisa Alchera, Matteo Monieri, Mirko Maturi, Irene Locatelli, Erica Locatelli, Silvia Tortorella, Angelina Sacchi, Angelo Corti, Manuela Nebuloni, Roberta Lucianò, Filippo Pederzoli, Francesco Montorsi, Andrea Salonia, Sandra Meyer, Jithin Jose, Pierangela Giustetto, Mauro Comes Franchini, Flavio Curnis, Massimo Alfano

**Affiliations:** a Unit of Urology, URI, Division of Experimental Oncology, IRCCS San Raffaele Scientific Institute, Milan, Italy; b Tumor Biology and Vascular Targeting Unit, Division of Experimental Oncology, IRCCS San Raffaele Scientific Institute, Milan, Italy; c Department of Industrial Chemistry "Toso Montanari", University of Bologna, Bologna, Italy; d Università Vita-Salute San Raffaele, Milan, Italy; e Pathology Unit, Department of Biomedical and Clinical Sciences, L. Sacco Hospital, Università degli Studi di Milano, Milan, Italy; f Department of Pathology, IRCCS San Raffaele Hospital and Scientific Institute, Milan, Italy; g FUJIFILM Visualsonics Inc., Amsterdam, the Netherlands; h Freedom Waves s.r.l., Milan, Italy

**Keywords:** Tumor targeting, Bladder cancer, Gold nanorods, Photoacoustic imaging, Early diagnosis

## Abstract

Detection and removal of bladder cancer lesions at an early stage is crucial for preventing tumor relapse and progression. This study aimed to develop a new technological platform for the visualization of small and flat urothelial lesions of high-grade bladder carcinoma in situ (CIS).

We found that the integrin α5β1, overexpressed in bladder cancer cell lines, murine orthotopic bladder cancer and human bladder CIS, can be exploited as a receptor for targeted delivery of GNRs functionalized with the cyclic CphgisoDGRG peptide (*Iso4*). The GNRs@Chit-*Iso4* was stable in urine and selectively recognized α5β1 positive neoplastic urothelium, while low frequency ultrasound-assisted shaking of intravesically instilled GNRs@Chit-*Iso4* allowed the distribution of nanoparticles across the entire volume of the bladder. Photoacoustic imaging of GNRs@Chit-*Iso4* bound to tumor cells allowed for the detection of neoplastic lesions smaller than 0.5 mm that were undetectable by ultrasound imaging and bioluminescence.

## 1 Introduction

Bladder cancers (BC) confined to the mucosa and invading the lamina propria are classified as stage Ta and T1, respectively, according to the Tumour, Node, Metastasis (TNM) classification system [1]. Intra-epithelial, high-grade tumors confined to the mucosa are classified as carcinoma in situ (CIS). Approximately 75% of patients with BC present with a disease confined to the mucosa (stage Ta, CIS) or submucosa (stage T1) [2]. All of these tumors can be treated by transurethral resection of the bladder (TURB), eventually in combination with intravesical instillations and are grouped as non-muscle invasive bladder cancer (NMIBC) for therapeutic purposes.

Bladder CIS is characterized by a small number of high-grade neoplastic cells that create a reddish area, indistinguishable from inflammation, and have a flat appearance in the urothelium. It can be missed or misinterpreted as an inflammatory lesion during cystoscopy if not biopsied. The management of patients with bladder CIS still represents a challenge in the onco-urological field [3], [4].

Several imaging methods such as computed tomography urography (CT urography), intravenous urography (IVU), ultrasound (US), multiparametric magnetic resonance imaging (mpMRI) and cystoscopy have been used to attempt the diagnosis of bladder cancer. However, major remaining limitations of diagnostic imaging involve the size of the tumor and the dimensional limit of detectability of any method, with US and CT resulting in very poor detection rates for bladder cancers < 5 mm in size [5]. Cystoscopy remains the gold standard diagnostic method for patients with the suspicion of bladder cancer [6]. Indeed, even cystoscopy, including photodynamic diagnosis performed using violet light after intravesical instillation of 5-ALA or hexaminolaevulinic acid, has limited diagnostic utility for CIS. In fact, during cystoscopy and TURB several biopsies from suspicious urothelium should be usually taken to detect and diagnose CIS on surgical tissue specimens [3]. Still, residual high-grade lesions are found in 40% of patients after the first TURB [7]. Due to these technological limitations, patients with bladder CIS experience very high frequencies of relapse following the first diagnosis, thus undergoing frequent and endless follow-up with poorly effective treatments, resulting in a poor quality of life and the highest cost per patient among all cancers [8].

To overcome the limitations of the imaging methods currently used in clinics for bladder CIS, we aimed to develop approaches and technologies for a non-invasive early diagnosis of in vivo orthotopic bladder cancer, by exploiting an imaging modality based on the photoacoustic (PA) imaging (PAI) approach. PAI is a hybrid imaging modality that combines the high contrast of optical absorption and the high spatial resolution of US generated by chromophores after irradiation by a non-ionizing pulsed laser. As acoustic waves generally undergo less scattering and tissue attenuation compared to light, PAI can provide higher resolution images than traditional US, and achieve deeper penetration than purely optical imaging systems [9], [10]. PAI also allows for the collection of functional and molecular information in real time by employing non-ionizing radiation to reach clinically relevant imaging depths [11].

Endogenous contrast agents, such as melanin, oxy and deoxy hemoglobin, lipids, collagen and water [12], and pulsed laser light in the near-infrared (NIR) spectral range have been exploited in PAI of melanoma [13], the tumour microenvironment [14], atherosclerotic plaque [15] and injuries [16], respectively. Exogenous contrast agents can also be used to enhance the sensitivity and spectroscopic specificity of PA signals. Targeted contrast agents can also be exploited to extend the range of applications of PAI to molecular imaging [17], [18]. Among the various contrast agents developed so far, gold nanoparticles are of particular interest for their versatility, unique optical and physicochemical properties, relatively inert nature, and successful use in many biomedical applications. In particular, gold nanorods (GNRs) show the highest extinction coefficient in the NIR range and high PA conversion efficiency. Furthermore, tuning the shape of GNRs allows the best wavelength of light stimulation to be selected, thereby enabling the use of these nanoparticles for the needed PAI application [17].

Integrins represent a potential target for human bladder cancer, because they are involved in almost every step of cancer progression from the primary tumor to late stage metastasis development [19]. Among the various integrins that play a role in cancer progression [20], we investigated the expression of the α5β1 integrin, whose overexpression has been reported in high-grade bladder cancer [21], [22] and as a marker of unfavorable prognosis for BC patients [23], [24].

Based on these premises, we have developed a new technological platform based on the use of i) GNRs that have been chemically engineered with chitosan (Chit) and the peptide *Iso4* (head-to-tail cyclized c(CphgisoDGRG) peptide, selective for the α5β1 integrin (*ki*=15 nM) [25]) to enable tumor targeting; ii) the intravesical instillation of urine-stable targeted GNRs (called GNRs@Chit-*Iso4*); iii) a technique of US-assisted shaking of GNRs@Chit-*Iso4,* to prevent nanoparticle sedimentation in the bladder; and, iv) the multimodal imaging of cancer lesions with PAI. We show that this platform is feasible and that it can be used to detect orthotopic murine bladder cancer lesions < 0.5 mm, undetectable by US imaging and bioluminescence.

## 2 Materials and Methods

### 2.1 Reagents

Bovine serum albumin (BSA) fraction-V, lipopolysaccharide from *Escherichia coli* O111:B4, and all the other reagents, if not specified, were from Sigma Aldrich (St. Louis, MO). Medical grade, endotoxin-free, chitosan prepared from Snow Crab (deacetylation degree 87.6% and viscosity 66 mPa.s at 20 °C) was from ChitoLytic (St. John's, Newfoundland, Canada). Maleimide-PEG_12_-NHS (1-maleimide-3-oxo-7,10,13,16,19,22,25,28,31,34,37,40-dodecaoxa-4-azatritetracontan-43-oic acid succinimidyl ester, 99%) was from Iris Biotech GmbH (Marktredwitz, Germany). Wild-type human integrin α5β1 (octyl β-D-glucopyranoside preparation) was from Immunological Sciences (Rome, Italy). Head-to-tail cyclized peptides c(CphgisoDGRG) and c(CGARAG), called *Iso4* and ARA, respectively, were from Biomatik (Delaware, USA). The identity and purity of *Iso4* and *ARA* were confirmed through mass spectrometry and HPLC analyses (expected/found monoisotopic mass, MH^+^, Da/Da, were 622.35/622.40 and 516.23/561.23, respectively; purity >95% in both cases). Peptides were dissolved in sterile water and stored in aliquots at − 80 °C. Human serum albumin (HSA) was from Baxter (Deerfield, IL). The *Iso4*-HSA conjugate (*Iso4* chemically coupled to HSA via 4-(N-maleimidomethyl)cyclohexane-1-carboxylic acid 3-sulfo-N-hydroxysuccinimide ester sodium salt (sulfo-SMCC)), and *HSA (HSA activated with sulfo-SMCC and quenched with β-mercaptoethanol instead of *Iso4*) were prepared as described [26].

### 2.2 Functionalization of GNRs@Chit with *Iso4* or Cys

GNRs@Chit was prepared from cetyltrimethylammonium bromide (CTAB)-coated GNRs (GNRs@CTAB; synthesis and characterization detailed in the Supplementary Information). To functionalize GNRs@Chit with *Iso4,* 30 ml of Maleimide-PEG_12_-NHS (1 mg/ml in ultrapure water, 35 µmol = 29.7 mg) were mixed with 30 ml of GNR@Chit (1 mM of Au, 30 µmol = 5.909 mg Au) under stirring to achieve a final weight ratio of Maleimide-PEG_12_-NHS:Au = 5:1. The mixture was then left to incubate overnight at room temperature and then dialyzed against ultrapure water using a 3.5 kDa cut-off dialysis tube for 24 h, at room temperature, to remove the excess crosslinker. The product (65 ml), consisting of activated GNRs@Chit-PEG_12_-maleimide, was then mixed with the *Iso4* peptide (21 mg, 34 µmol, 10 mg/ml in ultrapure water) and left to react for 24 h at room temperature. The unreacted maleimide groups were then quenched by adding an excess of cysteine hydrochloride (18 mg, 102 µmol). The product, called GNRs@Chit-*Iso4*, was then dialyzed against ultrapure water, the Au content quantified and the product aliquoted in vials containing 50 µg of Au was freeze-dried and stored at − 80 °C. In parallel, control nanoparticles with cysteine instead of *Iso4* were prepared as described above, but using an excess of cysteine (36 mg, 204 µmol) instead of the *Iso4* peptide. This product was called GNRs@Chit-Cys. About 50 vials of both products were prepared.

### 2.3 Characterization of the physicochemical properties of GNRs@CTAB, GNRs@Chit and functionalized GNRs@Chit-*Iso4.*

*Gold concentration* was determined by flame atomic absorption spectroscopy (FAAS) using a SpectraAA 100 Varian spectrometer (Agilent Technologies, Santa Clara, USA). Gold nanorods (100 μl) were dissolved in aqua regia (3 ml) and diluted to 10 ml with ultrapure water prior to analysis. For the calibration of the FAAS analysis, Au standard solutions at 1, 2, 5 and 10 mg/L were prepared by diluting the appropriate amounts of 1000 mg/ml TraceCERT® solutions in 30% aqua regia.

*VIS-NIR* absorption spectra (range 400–1100 nm) were recorded using a Cary 3500 UV–VIS–NIR modular spectrometer (Agilent Technologies, Santa Clara, USA) using a 1 cm path-length plastic cuvette.

*Transmission Electron Microscopy* (TEM) was performed using a TEM/STEM FEI TECNAI F20 operating at 200 keV equipped with a probe for energy-dispersing x-ray spectroscopy (EDX), selected-area electron diffraction (SAED) and High Angle Annular Dark Field Detector (HAADF). Before the analysis, samples were placed on a continuous-carbon film, supported on a copper grid and dried at 120 °C.

^*1*^*H NMR spectra* were obtained using a Varian Inova NMR spectrometer (14.09 T, 600 MHz). The chemical shifts were reported in ppm of frequency relative to the residual solvent signals (^1^H NMR: 4.80 ppm for heavy water).

*Viscosity measurements* were performed using an MCR102 (Anton-Parr, Graz, Austria) modular compact rheometer with a DPP25-SN0 geometry, i.e. a double plate geometry with a diameter of 25 mm.

*Zeta potential measurements* were performed using a Zetasizer-nano-S (Malvern Panalytical, Malvern, UK) in DTS1060C-Clear disposable zeta cells, at 25 °C.

*Thermogravimetric analysis* (TGA) was performed using a Q600 thermoscale (TA Instruments, New Castle, USA) working in nitrogen atmosphere from room temperature to 600 °C with a heating ramp of 20 °C/min, then switched to air and kept at 600 °C for 15 min

### 2.4 Limulus amebocyte lysate (LAL) assay

Endotoxin quantification was carried out using the Endotoxin Detection kit according to the manufacturer’s instructions (LAL QCL-1000, Lonza, Walkersville, MD).

### 2.5 Stability of GNRs@Chit and GNRs@Chit-*Iso4* in human urine

The stability of GNRs@Chit-*Iso4* in human urine was assessed by diluting the GNR solution (0.725 mM of Au in 10 ml of water) with human urine samples (10 ml) collected from 10 healthy adult volunteers (4 males and 6 females) and placed under stirring in a water bath at 37 °C. At various time points, 1 ml of the mixture was withdrawn, diluted in 10 ml of cold water (+ 4 °C) and subjected to VIS-NIR. A urine solution (50% in water) was used as the blank reference in the spectrometric analysis.

### 2.6 Cell lines

Two human primary bladder epithelial cells (ATCC catalog number PCS-420–010; CELLnTEC catalog number HBLAK) were cultured according to the manufacturer's instructions and used at passage four. Human bladder cancer cell lines RT4, 5637, and HT-1376 were from ATCC (catalog number HTB-2™, HTB-9™, HTB-4™, CRL-1472™, respectively), RT112 cell line was from Merck (catalog number 85061106). These cell lines were cultured in RPMI medium (Gibco; Thermo Fisher Scientific) with standard supplements. Murine bioluminescent MB49-Luc cells were kindly provided by Prof. Carla Molthoff (VU University Medical Center, The Netherlands) and cultured in DMEM medium (Gibco; Thermo Fisher Scientific) with standard supplements. For this cell line, a cell bank was prepared and authenticated for lack of cross-contamination by analyzing 9 short tandem repeats DNA [27] (IDEXX Bioanalytics, Ludwigsburg, Germany). A vial of cell bank was used to start a new experiment. The cells were routinely tested for mycoplasma contamination and cultured for not more than 4 weeks before use.

### 2.7 Human data collection

Human data collection followed the principles outlined in the Declaration of Helsinki. Patients signed an informed consent agreeing to supply their own anonymous information and tissue specimens for future studies. The study was approved by the Institutional Review Board (Ethic Committee IRCCS Ospedale San Raffaele, Milan, Italy). All methods were carried out in accordance with the approved guidelines.

Surgical specimens were staged according TNM classification [28]. Paired non-tumoral and tumoral bladder areas were from the same bladder of an individual submitted to TURB or radical cystectomy for bladder cancer; six TURBs with a histological diagnosis of CIS and two bladders with non-muscle invasive bladder cancer (NMIBC; CIS, pTa, pT1) and muscle invasive bladder cancer (MIBC; pT2-pT4) were used.

### 2.8 Immunohistochemical analysis

Tissue samples, fixed in 10% buffered formalin for 24–48 h at room temperature and then embedded in paraffin, were stained as previously reported [29]. Briefly, 3 µm tissue sections were dehydrated according to standard procedures and boiled twice in 0.25 mM EDTA pH 8.0 for 5 min using a microwave oven (780 W). After washing with PBS, and quenching the endogenous peroxidase with 3% H_2_O_2_, tissue sections were incubated with anti-α5 or -β1 rabbit monoclonal antibodies (Supplementary Table S1) for 2 h at room temperature. After washing, the binding of rabbit primary antibodies was detected using the Universal HRP-Polymer Biotin-free detection system (MACH4, BioCare Medical, USA) and 3,3-diaminobenzidine free base (DAB) as a chromogen. Tissue samples were then counterstained with Harris’ hematoxylin.

### 2.9 FACS analysis

The staining of α5 and β1 integrins expressed on the cell surface was carried out as described [30] using 5 µg/ml of the monoclonal antibodies listed in Supplementary Table S2**.** Isotype-matched antibodies were used as negative controls. The binding of primary antibodies was detected using Alexa Fluor 488-labeled goat anti-mouse or -hamster secondary antibodies according to their animal species.

### 2.10 Binding assays of *Iso4*-Qdot to MB49-Luc and 5637 cell lines

*Iso4* and ARA were coupled to amino-modified quantum dots nanoparticles, Qdot_605_ ITK Amino PEG (Thermo Fischer) as previously described [30]. The binding of *Iso4*-Qdot and ARA-Qdot to MB49-Luc and 5637 cells was assessed through FACS and fluorescence microscopy experiments. FACS analysis was carried out as follows: the cells were detached with DPBS containing 5 mM EDTA pH 8.0 (DPBS-E), washed with DPBS, and suspended in 25 mM Hepes buffer, pH 7.4, containing 150 mM sodium chloride, 1 mM magnesium chloride, 1 mM manganese chloride_,_ 1% BSA (*binding buffer-1*) and *Iso4*- or ARA-Qdot (range 30–0 nM, 5 ×10^5^ cells/100 μl tube). After 2 h of incubation at 37 °C, the cells were washed with *binding buffer-1* without BSA and fixed with 4% formaldehyde. Bound fluorescence was detected using a CytoFLEX S cytofluorimeter (Beckman Coulter). The binding of *Iso4*-Qdot and ARA-Qdot to living 5637 cells was analyzed as follows: 5637 cells were cultured in a 96-well clear bottom black plate (5 ×10^4^ cells/ well) for 48 h, 5% CO_2_, 37 °C. The plates were washed with *binding buffer-1* and incubated with *Iso4*-Qdot or ARA-Qdot solution (30 nM in *binding buffer-1*) for 2 h at 37 °C, 5% CO_2_. The cells were then washed with *binding buffer-1*, and fixed with 3% paraformaldehyde and 2% sucrose for 20 min. Bound fluorescence was acquired using the Cellomics ArrayScan XTI Studio Scan (Thermo Fischer Scientific) system.

### 2.11 α5β1 integrin binding assay

*Iso4* and ARA were chemically conjugated to maleimide-activated HRP (InnovaBioscience, cat. 401–0002), via thiol group, as follows: a vial of the lyophilized product was suspended in 1 ml of PBS (10 mM sodium phosphate buffer, pH 7.4, 138 mM sodium chloride, 2.7 mM potassium chloride, Sigma, P-3813) containing 5 mM EDTA (PBS-E); the solution was then divided into 2 aliquots and mixed with *Iso4* or ARA peptide, using a 3:1 peptide: enzyme ratio (mol/mol). The final products were called *Iso4*-HRP and ARA-HRP. The conjugates were diluted (range 0–400 nM) in 25 mM Tris-HCl, pH 7.4, containing 150 mM sodium chloride, 1 mM magnesium chloride, 1 mM manganese chloride and 1% BSA (*binding buffer-2*) and added to 96-well plate polyvinyl chloride (PVC) microtiter plates (Carlo Erba, cod. FA5280100) coated with or without α5β1 (4 μg/ml) and left to incubate for 2 h. The plates were washed with 25 mM Tris-HCl, pH 7.4, containing 150 mM of sodium chloride, 1 mM of magnesium chloride, 1 mM of manganese chloride, and bound peroxidase was detected by adding a chromogenic solution (o-phenylenediamine dihydrochloride, OPD). The chromogenic reaction was stopped by adding 1 N sulfuric acid. The absorbance at 490 nm was then measured using a microtiter plate reader. The effect of human urine (from a healthy donor) on the binding of *Iso4*-HRP was studied as described above by mixing *Iso4*-HRP (300 nM final concentration) and various amounts of urine diluted in *binding buffer-2*.

### 2.12 Cell adhesion assays

96-well PVC microtiter plates were coated with *Iso4*-HSA or GNRs@Chit-*Iso4* in 50 mM sodium phosphate, pH 7.3, containing 150 mM sodium chloride, (overnight incubation at 4 °C). The plates were washed and blocked with 2% BSA in DMEM or RPMI-1640 (200 μl/well, 1 h). MB49-Luc and 5637 cells were detached with DPBS-E, washed twice with DPBS, and suspended in DMEM or RPMI-1640 containing 0.1% BSA (*binding buffer-3*), and added to the coated-plates (1.5 ×10^5^ cells/well, 100 μl). After 1–2 h of incubation at 37 °C, 5% CO_2_, the plates were washed with *binding buffer-3.* Adherent cells were fixed and stained with 0.5% crystal violet in 20% methanol. After washing with water, the dye was extracted from cells using a 10% acetic acid solution (140 μl/well) and the absorbance at 570 nm was measured using a microplate reader. The effect of urine on the pro-adhesive properties of GNRs@Chit-*Iso4* was investigated as described above, except that the cells were seeded in *binding buffer-3* or *binding buffer-1* and various amounts of human urine (obtained from a healthy volunteer).

### 2.13 Quantification of *Iso4* peptide bound to GNRs@Chit

The amount of *Iso4* loaded on GNRs@Chit-*Iso4* was quantified by measuring the total aminoacidic contents after acidic hydrolysis of the nanoparticles by Alphalyse Inc., Denmark.

### 2.14 Murine orthotopic bladder tumor model

All procedures and studies involving mice were approved by the Institutional Animal Care and Use Committee of San Raffaele Scientific Institute and performed according to the prescribed guidelines (IACUC, approval number 942). Female albino C57BL/6 J mice (9 weeks old, weighing about 20 g, Charles River Laboratories, Italy) were anesthetized with ketamine (80 mg/kg) and xylazine (15 mg/kg) and kept in dorsal position. Using a 24-gauge catheter, the bladder of each mouse was emptied and instilled with or without MB49-Luc cells (10^5^ cells/100 μl in DPBS). Thirty minutes later the catheter was removed, and mice were allowed to recover and returned to their cage. Tumor growth was monitored by measuring the tumor volume through US imaging (see below) and in vivo bioluminescent quantification after administration of luciferin (15 mg/kg, intra peritoneum) using the non-invasive In Vivo Imaging System (IVIS, PerkinElmer, USA).

### 2.15 US and PAI of GNRs@Chit and GNRs@Chit-*Iso4.*

High-resolution US and PA imaging have been acquired using the Vevo LAZR-X platform (FUJIFILM VisualSonics, Inc.,Toronto, ON, Canada). The imaging platform includes a high frequency US system (Vevo 3100) combined with an Nd:YAG nano-second pulsed laser with repetition rate of 20 Hz. The linear US transducer array Mx 550D consists of 256 elements with a nominal center frequency of 40 MHz (25–55 MHz bandwidth) and a spatial resolution of 40 µm with a maximum imaging depth of 15 mm. Light from the laser is delivered to the tissue through optical fibers mounted on either side of the transducer. During volumetric US-PA acquisitions, a stepper motor is used for the linear translation of the US transducer and optical fibers along the sample. The linear stepper motor moves in steps of a minimum of 0:1 mm while capturing 2-D parallel images, for a maximum 3D range distance of 6.4 cm.

3D B mode scan was carried out for in vitro (drop) and in vivo (mouse bladder) studies.

The photoacoustic spectra between 680 nm and 970 nm were scanned with a step size of 5 nm; for the in vivo studies (murine bladder) the 3D multispectral PA scans were acquired selecting PA spectral curve of tissue components melanin, deoxy- and oxy-genated blood, and GNRs; the processed wavelengths (680; 722; 764; 810; 924; 970 nm) were automatically selected from the spectral curve used to spectrally unmix the GNRs signal from other endogenous tissue chromophore signals such as oxy, deoxy hemoglobin. The algorithm reported by Luke et al. [31] was used to select these wavelengths which is ideal for separating the signal from GNRs from other endogenous absorbers. For the in vitro studies (agar drops embedded in slime) the 3D multispectral PA scans were acquired selecting the PA spectral curve of slime and GNRs (processed wavelengths 680; 782; 810 nm).

Data analyses were conducted using the Vevo®Lab software; volumes of interest were obtained by manually drawing Volumes of Interest (VOIs) on 3D B-mode images. GNRs, melanin, oxy- and deoxy-hemoglobin content were estimated through spectral unmixing analyses of the spectroscopic data.

*Light attenuators.* Since the GNRs are susceptible to change in shape at the higher laser threshold, light attenuators were prepared to reduce the laser fluence to avoid GNRs reshaping. Light attenuators were prepared as follows: agar powder (cat. A9539, Sigma) was suspended in deionized and distilled water (1% final concentration), melted at 95 °C, and mixed with different concentration of Intralipid (cat. I141, Sigma). The mixtures (3 ml) were poured into Disposable Base Molds (30×24×5 mm, Bio-Optica, Milan, Italy) (Supplementary Fig. S1A), left to solidify for 2 min at room temperature, and stored in a humidified chamber until used. The light attenuator was then sliced and mounted in contact with the optical fibers.

*In vitro* PAI of GNRs was carried out as follows: GNRs (30 μl in DPBS with calcium and magnesium) were mixed with a 1% agar solution (30 μl), the mixture was then poured on Parafilm® M (Sigma) and left to solidify in a humidified chamber (Supplementary Fig. S1B). The solidified product (called “agar drop”) was then placed on an ultrasound gel pad (Aquaflex, Parker), embedded in slime made of polyvinyl alcohol polymer crosslinked with sodium tetraborate (Barrel of slime) (Supplementary Fig. S1C) and PAI was performed using the two optical fiber bearing light attenuators. The transducer was perpendicularly placed on the object under investigation previously covered with ultrasound transmission gel (Aquasonic 100, Parker). Axial sections were acquired using the following settings for B-Mode: 2D Power 100%; 2D Gain 13 dB; for PA-mode: Pa Power 100%; PA gain *44 dB*; TGC and depth were maintained identical for all drops. PA and US data have been analyzed using VevoLab 3.2.5 software (Supplementary Fig. S1D).

*Light fluence.* The energy of the laser at the wavelength of 750, 800 and 850 nm was measured for 2 min using a laser energy meter (PE50BF-DIFH-C, *P*/*N 7Z02943,* Ophir, Germany). The laser beam size was assessed by shooting the laser for 5 s onto a piece of photographic paper (Kodak Linagraph Type 1895, Eastman Kodak Company, New York, USA) placed 8 mm from the light source. The resulting burned area was then quantified with a ruler (Supplementary Fig. S1E**)** and the light fluence was calculated by dividing the light energy by the light beam size.

*Low frequency US-assisted shaking of GNRs and* in vivo *PAI.* GNRs (100 μl equivalent to 3 nmol) were instilled in the murine bladder that was identified by US imaging using the MX550D transducer placed perpendicular to the abdomen. Next, the shaking was performed by using the in vitro medical device Waver designed and produced by Freedom Waves, composed by a piezoelectric matrix array transducer (cod. 2048P1002, manufactured by Vermon, France) connected to a digital ultrasound power supply and amplifier manufactured by SYES, Italy. Mice were exposed to 3 cycles of shaking (3 min of shaking followed by 1 min of stop) using 1 MHz ultrasound with a duty cycle of 20% and 51 Joule of energy. After the shaking period the bladder was emptied from GNRs and washed twice with DPBS, and the PA acquisitions were performed. Axial sections were acquired using the following settings for B-Mode: 2D Power 50%; 2D Gain 23 dB; for PA-mode: Pa Power 100%; PA gain *39 dB*. TGC and depth were maintained identical for all acquisitions. All imaging experiments on mice were conducted under gaseous anesthesia (Isoflurane/air 4% for induction and 1.5% thereafter).

*Velocity and volumetric flow rate of the GNRs.* The velocity and volumetric flow rate of the bladder content (i.e. instilled with or without GNRs) was measured by simultaneously placing the piezoelectric matrix array transducer and the MX550D transducer on the abdomen of a mouse, at 45 degrees from each other (Supplementary Fig. S2). B-mode US (300 frames equal to 5 s) was acquired at 25% of transmitted power, to reduce the impact of the ultrasound wave on the velocity and distribution of the GNRs. Acquisitions were taken in the median part of the bladder corresponding to the section with the largest size and volume. Acquired Avi data were then used for particle image velocimetry analysis using the PIVlab 2.45 software [32].

*Simulation of the energy transport in the murine bladder.* A numerical simulation of the light fluence was performed to estimate the energy distribution within the tissue domain. The light energy distribution was obtained by implementing the Monte Carlo model of light transport, based on the MCXLAB computer simulation. The optical properties (the absorption coefficient (µ_a_), the scattering coefficient (µ_s_), the scattering anisotropic factor (*g*), and the refractive index (*n*)) of the skin, tissue surrounding the bladder, urine and GNRs used for the simulation were recently described [33], and reported in Supplementary Table S3. Fluence simulation was carried out with $10^{9}$ photons at 800 nm, considering a Gaussian light source within a 120 × 120 pixel domain of tissue.

## 3 Results

### 3.1 Human bladder CIS, but not non-neoplastic bladder epithelium, expresses α5β1 integrin

**Fig. 1:**
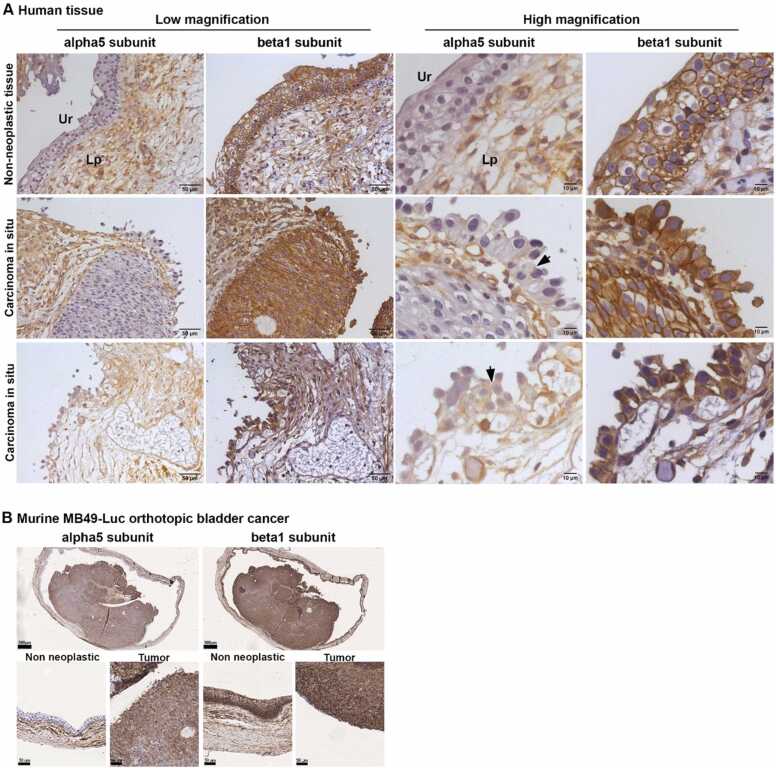
**Expression of α5 and β1 integrins in human bladder CIS, and murine non-invasive orthotopic bladder cancer. A**) Representative immunohistochemistry photomicrographs of human bladder sections of non-neoplastic tissue and CIS obtained by TURB; one representative image of normal tissue and two representative images of CIS are shown. Tissues were immunostained with the indicated anti-integrins antibodies. Ur; Urothelium, Lp; Lamina propria. Arrows indicate the membrane stain of the α5 integrin. **B**) Representative immunohistochemistry photomicrographs of murine orthotopic bladder cancer, 11 days after the intravesical instillation of MB49-Luc cells, and immunostained with the indicated anti-integrins antibodies. One murine bladder out of the 3 analyzed is shown. Upper panels show low magnification (scale bar 500 µm); lower panels show higher magnification of non-neoplastic and neoplastic tissues (scale bar 50 µm).

The expression of the α5β1 integrin in non-neoplastic and neoplastic bladder tissues was evaluated through immunohistochemical analysis of tissue sections obtained from TURB specimens with a histological diagnosis of CIS. The α5 subunit was not expressed by the non-neoplastic urothelial cells, while membrane staining was observed in the CIS; stromal cells in the lamina propria of non-neoplastic and neoplastic bladder tissues showed similar expression (Fig. 1**A**). The β1 subunit was strongly expressed by non-neoplastic urothelial cells and stromal cells, as well as by the CIS (Fig. 1**A**). Lack of α5 subunit expression in the normal urothelium was also observed in the Von Brunn’s Nests and the expression of the α5 subunit in CIS was confirmed in five out of six TURBs (Supplementary Fig. S3), while the β1 subunit was always expressed by the normal urothelium and stromal cells. In one out of two radical cystectomies we confirmed the expression of the α5 subunit in the CIS and a zonal expression in non-infiltrating papillary, while little or no expression was observed in the infiltrating tumor pT1, pT2 and pT4 (Supplementary Fig. S4). Therefore, α5β1 might represent a potential receptor for targeting non-infiltrating tumors such as the non-infiltrating papillae and CIS.

Next, we checked the expression of the α5β1 integrin on two non-tumoral human primary urothelial cells (PCS-420–010 and HBLAK) and on four bladder cancer cell lines (RT4, RT112, 5637 and HT1376) derived from human tumors of different stage and grade [34] through flow cytometry analysis using a different set of anti-integrin antibodies. The results showed that human bladder cancer cell lines express more α5 and β1 than human primary urothelial cells (Supplementary Table S4). Taken together these results suggest that the expression of α5β1 by human bladder CIS may represent a potential target for the development of new tumor targeted diagnostic tools based on α5β1 targeting.

### 3.2 MB49-Luc murine orthotopic bladder tumor, but not normal bladder epithelium, expresses α5β1

We investigated whether MB49-Luc tumor bearing-mice, a widely used syngeneic model in orthotopic bladder cancer [35], could recapitulate the α5β1 expression pattern observed in the human bladder CIS. The results showed that the α5 subunit was expressed by MB49-Luc tumor cells, but not by the non-neoplastic adjacent epithelial cells. In contrast, stromal cells of both neoplastic and non-neoplastic tissues showed a similar level of expression (Fig. 1**B)**. The β1 subunit was expressed by the basal urothelial cells in the non-neoplastic and neoplastic tissue (Fig. 1**B**). Accordingly, flow cytometry analysis confirmed that MB49-Luc cells express the α5β1 integrin (Supplementary Table S5).

Therefore, mice bearing the MB49-Luc derived orthotopic tumor could be used as a preclinical model for the development of new tumor targeted strategies based on α5β1 targeting.

### 3.3 MB49-Luc and 5637 cells can be efficiently targeted by *Iso4* peptide

**Fig. 2:**
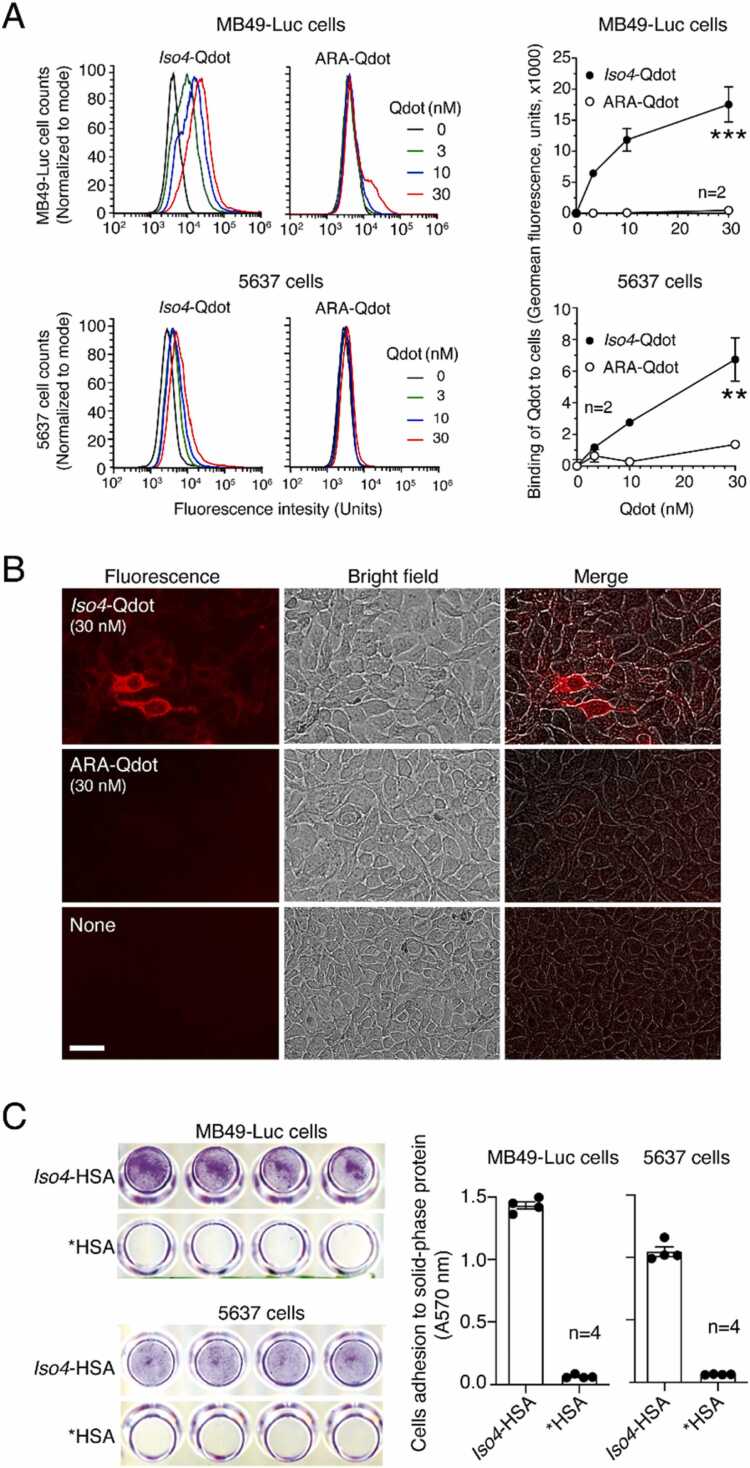
**Binding of*****Iso4*****peptide to MB49-Luc and 5637 cell lines. A**) Binding of *Iso4*-Qdot or ARA-Qdot (control) to MB49-Luc and 5637 cells as measured by FACS. A Representative FACS experiment (*left*) and quantification of Qdot binding (*right*) are shown. *Circles*: mean±SEM of duplicates. **B**) Binding of *Iso4*-Qdot or ARA-Qdot to living 5637 cells. Cells were grown in a 96-well plate and incubated with the indicated dose of Qdot (for 2 h, 37 °C, 5% CO_2_)_,_ after washing and fixing cell bound fluorescence was acquired using the Cellomics ArrayScan XTI Studio Scan (Thermo Fischer Scientific) system. Magnification 20X; scale bar 10 µm; red, Qdot. **C**) Adhesion of MB49-Luc and 5637 cells to solid-phases coated with *Iso4*-HSA or *HSA (control) and stained with crystal violet. Representative images of wells coated with 30 µg/ml of *Iso4*-HSA or *HSA (*left*) and the quantification of cell adhesions (*right*). Images were acquired with a scanner. *Bars,* mean±SE, n = 4.

To assess whether *Iso4* can recognize α5β1-positive bladder cancer cells, we coupled *Iso4* to fluorescent nanoparticles (Quantum Dots, Qdot) via the sulfhydryl group of the cysteine, and evaluated the binding of this conjugate (*Iso4*-Qdot) to MB49-Luc and 5637 cells. An irrelevant head-to-tail cyclized c(CGARAG) peptide was also coupled to Qdot (ARA-Qdot) and used as a negative control. Flow cytometry and fluorescence microscopy experiments showed that *Iso4*-Qdot, but not the ARA-Qdot, bound these cells with a potency that correlates with the expression level of α5β1 on these cells (Fig. 2**A and B**).

Next, given that peptides containing isoDGR coupled to human serum albumin (HSA) can promote and support endothelial cell adhesion [25], [36], we coupled *Iso4* to HSA and tested the conjugate (*Iso4*-HSA) in cell adhesion assays using MB49-Luc and 5637 cells. The results showed that *Iso4*-HSA, but not the activated HSA lacking the peptide (*HSA), promoted and supported cell adhesion (Fig. 2**C**), suggesting that both cell lines express functional *Iso4* receptors, likely α5β1. Taken together, these results suggest that *Iso4* can be coupled with different compounds in a functional manner and exploited to target α5β1-positive bladder cancer cells.

### 3.4 Conjugation of *Iso4* to GNRs@Chit

**Fig. 3:**
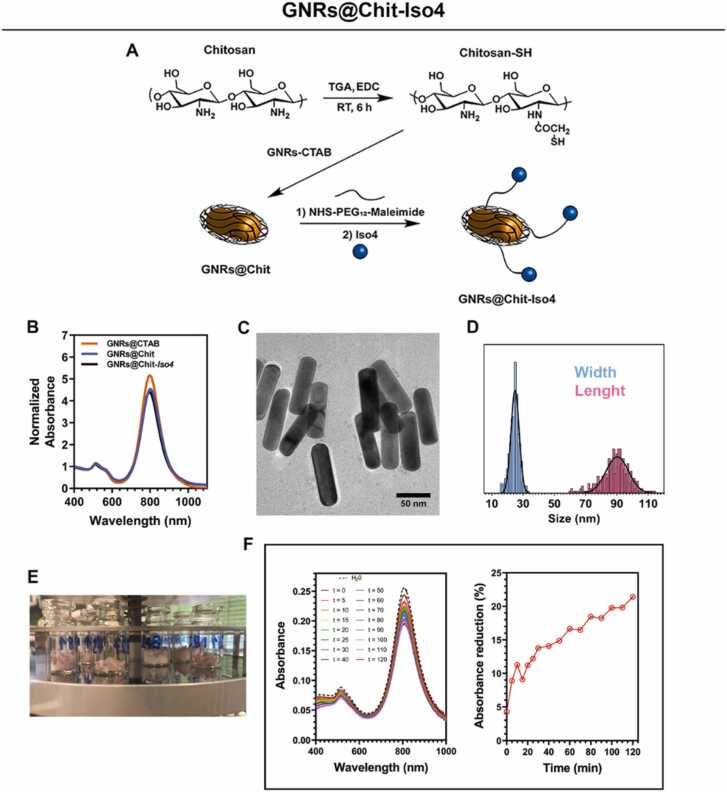
**Synthesis and characterization of GNRs@CTAB, GNRs@Chit and GNRs@Chit-*****Iso4*****. A**) Steps for the synthesis of GNRs@Chit-*Iso4*. Chemical modification of chitosan via EDC-coupled amidation of thioglycolic acid with chitosan amino groups; removal of CTAB by the thiolated chitosan that binds GNRs; attachment of the bifunctional PEG linker to the NHS ester terminus to the free amino groups on chitosan; conjugation of *Iso4* by exploiting its cysteine residue that is reactive towards the maleimide terminus of the linker. **B**) VIS-NIR spectra GNRs@Chit-*Iso4* compared to the VIS-NIR spectra of GNRs@CTAB and GNRs@Chit. **C)** Shape of GNRs@Chit-*Iso4* by TEM analysis (scale bar = 50 nm). **D**) Distribution of width and length of the nanorods present in the nanosystem GNRs@Chit-*Iso4*, and the corresponding Gaussian fit (mean ± SD and R^2^ for length 90.2 ± 7.2 nm and 0.86; mean ± SD and R^2^ for width 24.9 ± 2.6 nm and 0.93. n = 300 by TEM). **E**) Aliquots of GNRs@Chit-*Iso4* during the freeze-drying process. **F**) VIS-NIR spectra and absorption intensity of GNRs@Chit-*Iso4* in the presence of human urine over time.

To have GNRs on hand that can be exploited for the PAI of α5β1-positive tumors, we functionalized Chitosan-coated GNRs (GNRs@Chit) that were prepared from CTAB-coated GNRs (GNRs@CTAB) having a longitudinal surface plasmon resonance peak centred at 800 nm (detailed methods in Supplementary Material and Supplementary Fig. S5-S8). GNRs@Chit was functionalized with cyclo-CphgisoDGRG (*Iso4*), a selective ligand of the α5β1 integrin. To this aim, we exploited a heterobifunctional crosslinker reagent, composed of an ethylene oxide spacer (PEG_12_) bearing a *N*-hydroxysuccinimidyl (NHS) ester to its extremities and a maleimide functional group (NHS-PEG_12_-maleimide). The NHS ester terminus reacts with the free amino groups on chitosan ensuring the binding of the linker to the chitosan, while the maleimide group is available to react in a further step with the sulfhydryl group of *Iso4*. *Iso4* was then coupled with activated GNRs@Chit, and control GNRs with cysteine (Cys) in place of *Iso4* were also prepared. The resulting product was called GNRs@Chit-*Iso4* and GNRs@Chit-Cys (Fig. 3**A**). Next, we assessed the physical-chemical properties of the metal core of GNRs@Chit-*Iso4*, determining the longitudinal-LSPR to peak at 802 nm (Fig. 3**B**), shape and dispersion (Fig. 3**C**), and aspect ratio of 3.62 ± 0.67 (length 90.2 ± 7.2 nm, width 24.9 ± 2.6 nm, Fig. 3**D**). These findings show that conjugation of *Iso4* does not modify the physical-chemical properties of GNRs, with only small and gradual variation of the longitudinal-LSPR peak vs GNRs@Chit (800 nm) and GNR@CTAB (798 nm) that is representative of the variation of the chemical environment surrounding the GNRs. Both the Cys- and the *Iso4*-conjugated nanosystems displayed a reduction in the Z-potential (+ 10.3 mV and + 10.6 mV for GNRs@Chit-*Iso4* and GNRs@Chit-Cys, respectively, vs. + 40 mV measured for GNRs@Chit), according to the reduced amount of free amino groups on chitosan due to the presence of the PEG linker.

Moreover, the crystallinity and the composition of the GNRs@Chit-*Iso4* was proven to be conserved after the consecutive conjugation steps, additionally revealing no detectable Br atoms from CTAB (Supplementary Fig. S9A). GNRs@Chit-*Iso4* was therefore aliquoted in vials, freeze-dried and vacuum sealed to achieve 50 sterile single use vials containing the lyophilized product which results in a GNRs@Chit-*Iso4* concentration of 1 mM when dissolved in 500 μl of water (Fig. 3**E**).

The stability of GNRs@Chit-*Iso4* in human urine was then investigated at 37 °C for up to 2 h of incubation. No significant change in the shape of the LSPR spectrum occurred upon incubation in urine up to 2 h (Fig. 3**F**), with a 14% reduction of the absorbance at ∼800 nm observed in the first 40 min, and a further 7% reduction in the following 80 min of incubation (Fig. 3**F**). These results suggest that GNRs@Chit-*Iso4* can be incubated at 37 °C in urine for at least 2 h without a dramatic loss of its optical properties in the NIR. The same techniques were employed for the characterization of GNRs@Chit-Cys, revealing no appreciable discrepancies with respect to GNRs@Chit-*Iso4* (Supplementary Table S6**).**

### 3.5 Biochemical and biological characterization of GNRs@Chit-*Iso4* and GNRs@Chit-Cys

To quantify the amount of peptide loaded onto GNRs@Chit-*Iso4*, we measured the total amino acid composition present in the supernatant of GNRs@Chit-*Iso4* after acidic hydrolysis. Hydrolysed GNRs@Chit-Cys was used as a negative control since it is expected to be an “amino acid-free” compound (Supplementary Fig. S10). The results of the quantification of GNRs@Chit-*Iso4* revealed that ∼1 × 10^11^ nanoparticles are loaded with about ∼ 0.5 mg peptide, which corresponds to ∼ 6 × 10^6^ peptides per GNR (Supplementary Table S7).

**Fig. 4:**
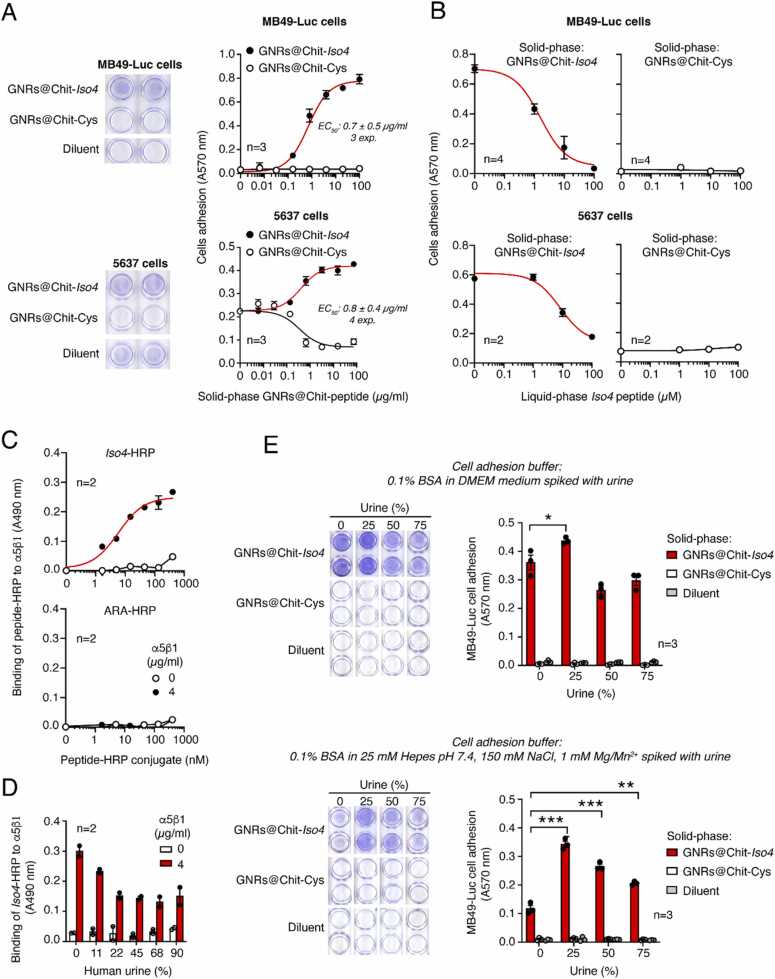
**GNRs@Chit-*****Iso4,*****but not GNRs@Chit, promotes MB49-Luc and 5637 cell adhesion and spreading. A)** Adhesion of MB49-Luc and 5637 cells to microtiterplates coated with various amounts of GNRs@Chit-*Iso4* or GNRs@Chit-Cys. Representative images of cell adhesion to 250 µg/ml of gold nanorods (according to dry matter content) and the quantification of cell adhesion are shown (*Circles*, mean±SE, n = 3). The EC_50_ values reported in each plot are the result of 3–4 independent experiments (mean±SE). **B**) Effect of free *Iso4* on MB49-Luc and 5637 cell adhesion to microtiterplates coated with 250 µg/ml of GNRs@Chit-*Iso4* or GNR@Chit-Cys. MB49-Luc and 5637 cells were mixed with the indicated amount of free *Iso4* and left to adhere to microtiter plates coated with GNRs. A representative experiment out of three independent experiments is shown (*Circles*, mean±SE, n = 2). **C**) Binding of various amounts of *Iso4*-HRP or ARA-HRP to microtiter plates coated with or without α5β1 as detected with OPD chromogenic substrate (*Circles*, mean±SE, n = 2).**D**) Effect of human urine on the binding of *Iso4*-HRP to microtiter plates coated with or without α5β1. *Iso4*-HRP (300 mM) was mixed with the indicated amount of urine, and the mixtures were added to the plates. After washing, bound peroxidase was detected by OPD chromogenic substrate (*Bars*, mean±SE, n = 2). **E**) Effect of human urine on MB49-Luc cell adhesion to solid-phase coated with GNRs@Chit-*Iso4* or GNRs@Chit-Cys. Cells were suspended in DMEM containing 0.1% BSA (***upper panel***) or in 25 mM Hepes buffer, pH 7.4, containing 150 mM sodium chloride, 1 mM magnesium chloride, 1 mM manganese chloride and 0.1% BSA (***lower panel***) and spiked with the indicated amounts of human urine. The mixtures were added to microtiter plates coated with 250 µg/ml of GNRs and left to incubate for 1–2 h at 37 °C, 5% CO_2_. After washing, the adherent cells were fixed and stained with crystal violet. Representative images of cell adhesion to wells coated with 250 µg/ml of gold nanorods and the quantification of cell adhesion are shown (*Bars*, mean±SE, n = 3). * , P < 0.05; * *, P < 0.01; * ** , P < 0.001 by two tailed *t*-test as determined using the GraphPad Prism software.

Next, to demonstrate the presence of functional *Iso4* on GNRs, we measured the capability of GNRs@Chit-*Iso4* and GNRs@Chit-Cys to promote cell-adhesion. To this aim, microtiter plates were coated with various amounts of GNRs@Chit-*Iso4* or GNRs@Chit-Cys and seeded with MB49-Luc and 5637 cells. GNRs@Chit-*Iso4*, but not GNRs@Chit-Cys, induced cell adhesion and spreading of both cell lines (Fig. 4**A**), suggesting that the conjugation of *Iso4* to GNRs@Chit does not affect the peptide function. Of note, the effective concentration 50 (EC_50_) of GNRs@Chit-*Iso4* for both cell lines was similar, being 0.73 ± 0.05 µg/ml for MB49-Luc cells and 1.3 ± 0.6. µg/ml for 5637 cells (Fig. 4**A**). The addition of an excess of free *Iso4* peptide completely inhibited cell adhesion, suggesting that the adhesion was specific and dependent on the *Iso4* peptide being coupled with GNRs (Fig. 4**B**).

### 3.6 Effect of urine on the binding of GNRs@Chit-*Iso4* to the α5β1 integrin and MB49-Luc cells

Intravesically administered GNRs@Chit-*Iso4* is expected to target bladder tumor cells in a harsh environment characterized by the presence of urine and a broad variety of metabolites [37], bacteria [38], bacteria-derived mucus and floating urothelial cells that might impair the binding capability of GNRs@Chit-*Iso4* to α5β1. With the intention of using targeted GNRs in the bladder with a protocol similar to that used for the photodynamic diagnosis performed using Hexvix [39], we envisaged bladder draining before the intravesical instillation of GNRs, thus expecting that instilled GNRs would mix with increasing amounts of urine over time.

Thus, we first investigated the impact of human urine on the binding of *Iso4* to purified α5β1. To this aim, *Iso4* was coupled with maleimide-activated horseradish peroxidase (HRP) to produce an *Iso4*-HRP conjugate to be used as a probe in direct binding assays with microtiter plates coated with α5β1. An HRP control conjugate using the ARA peptide instead of *Iso4* was also prepared (ARA-HRP). As expected, *Iso4*-HRP, but not ARA-HRP, could bind α5β1 in a dose dependent manner (Fig. 4**C**), suggesting that the probe is functional. Of note, to recapitulate the urine environment we omitted the addition of any detergent in the binding and washing buffers. The effects of various amounts of human urine (range 11.5 – 90%) were then tested. The results showed that urine partially inhibited the binding of *Iso4*-HRP, with the overall recovery of binding being about 55% in 90% of urine (Fig. 4**D**), suggesting that *Iso4* can still bind to α5β1 even in this unfavorable condition.

We further investigated the effects of human urine on the capability of GNRs@Chit-*Iso4* to bind to MB49-Luc cells. To this aim, the effects of various amounts of urine on MB49-Luc cell adhesion to GNRs@Chit-*Iso4*-coated plates was tested. In parallel, GNRs@Chit-Cys-coated plates were used as negative controls to monitor the unspecific cell adhesions. Surprisingly, when MB49-Luc cells were seeded in DMEM medium containing 25–75%urine, a significant increase (∼20%) of cell adhesion to GNRs@Chit-*Iso4*, but not to GNRs@Chit-Cys, or to the control plate (lacking nanoparticles), was observed (Fig. 4**E**). The gain of cell adhesion to GNRs@Chit-*Iso4* caused by the low concertation of urine was even more marked when the assays were carried in Hepes buffer supplemented with divalent ions (Mg^2+^/Mn^2+^) (Fig. 4**F**).

These results suggest that urine does not prevent the capability of *Iso4* grafted onto GNRs to bind to bladder cancer cells.

### 3.7 Set up for in vitro detection of the PA signal of GNRs

Since GNRs are susceptible to shape change and a consequent loss of photoacoustic properties when stimulated with pulsed light above a certain energy threshold, light attenuators were used to reduce laser fluence to avoid the reshaping of the GNRs. The in vitro PA properties of the GNRs were investigated using agar drops containing GNRs@Chit-*Iso4* or GNRs@Chit-Cys (15 nmol Au), and 0.6% Intralipid (IL) based light attenuators mounted on the tip of the optical fibers of the Vevo LAZR-X (Supplementary Fig. S1).

**Fig. 5:**
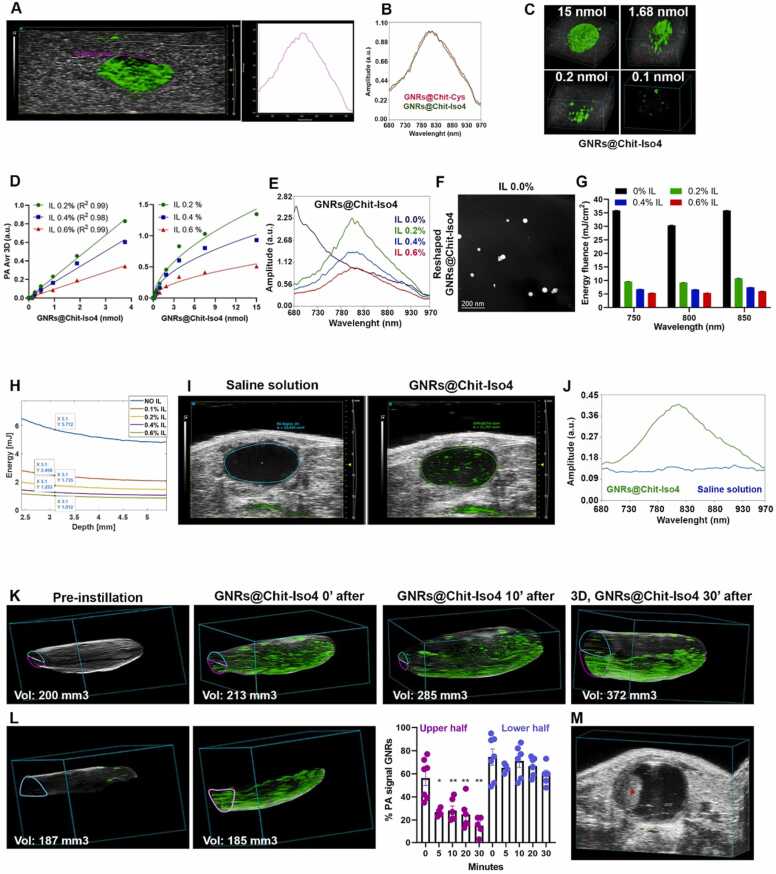
***In vitro*****and in vivo PAI of GNRs@Chit-*****Iso4*****. A**) PAI of agar drop containing GNR@Chit-*Iso4* (15 nmol Au) and its associated PA spectrum using 0.6% Intralipid (IL) light-attenuators. The echogenic signal (*gray*) is generated by the slime in which the agar drop is embedded. The violet *ROI* delineates the PA signal of GNRs from which the PA spectrum was derived; the PA images were acquired and spectrally unmixed with VevoLab software to separate the contribution of the slime and GNRs (the *green signal* corresponds to the PA signal of GNRs). **B**) Normalized PA spectra of GNRs@Chit-Cys and GNRs@Chit-*Iso4* (15 nmoli of Au) embedded in agar drop (one representative experiment of five). **C**) 3D distribution of the GNRs@Chit-*Iso4* signal in agar drops acquired using the light attenuators made of 0.6% IL. **D**) Dose-response plot of the PA signal of GNRs@Chit-*Iso4* in agar drop analyzed using light attenuators prepared with the indicated concentration of IL; linear dynamic range from 0 to 3.75 nmol Au, and trend towards plateau from 3.75 to 15 nmol Au (mean±SEM of duplicates are shown). **E**) Overlayed PA spectra of GNRs@Chit-*Iso4* (3.75 nmol Au) acquired using light attenuators prepared with the indicated concentration of IL. **F**) TEM analysis of GNRs@Chit-*Iso4* recovered from an agar drop after PA analysis conducted with a light attenuator prepared without IL. **G**) Energy fluence at 750, 800 and 850 nm, both in the absence and in the presence of light attenuators containing the indicated amounts of IL (mean±SEM of triplicates). **H**) Average energy distribution along the depth, from the Monte Carlo model of the simulated domain reported in the Supplementary Fig. S11. **I**) In vivo PAI of murine bladder after intravesical instillation of 100 μl of vehicles (saline solution) or GNRs@Chit-*Iso4* (3 nmol Au), from representative frames taken in the middle of the bladder (one representative experiment of five); the *green signal* corresponds to the PA signal of GNRs after unmixing the PA signal of melanin, deoxy- and oxy-genated blood and GNRs. **J**) PA spectra of the saline solution and of GNRs@Chit-*Iso4* (3 nmol Au) in the murine bladder. **K**) 3D distribution of the GNRs@Chit-*Iso4* (3 nmol Au) PA signal in murine bladder imaged at the indicated time points (one representative experiment of five). **L**) 3D distribution of the GNRs@Chit-*Iso4* PA signal in the upper and lower half of the murine bladder 30 min after instillation, followed by the quantification of the volume occupied by the PA signal (% PA signal of GNRs) in the upper and lower half of the bladder at the different time points (data shown as mean±SEM, each dot representative of one animal). * , * *; p value using 2-tailed Mann-Whitney test between upper and lower half of the bladder each time point). **M**) PAI of the murine bladder with a well-established tumor on the left side (red asterisk), showing a lack of PA signal after the intravesical instillation of GNRs@Chit-*Iso4* (3 nmol Au) and two intravesical washing steps to remove the unbound GNRs; axial diameter of the bladder lumen = 3.7 mm. Vol: Volume.

The results showed similar PA signals and spectrum for GNRs@Chit-*Iso4* or GNRs@Chit-Cys, with maximum peaks at 810 nm (Fig. 5**A and 5B**), and about 0.2 nmol of Au was detected using 0.6% IL (Fig. 5**C)**. Next, we assessed the minimum amount of IL required for the detection of the highest PA signal and the correct spectra of GNRs in vitro*.* Using light attenuators containing 0.2%, 0.4% and 0.6% IL, we found that the PA signal intensity of GNRs@Chit-*Iso4* was inversely proportional to the amount of IL (Fig. 5**D**). As expected, GNRs@Chit-*Iso4* analyzed using light attenuators without IL underwent reshaping, as observed by the change of shape of the PA spectrum (Fig. 5**E)** and further supported by the transition from the rod-like (Fig. 3) to a spherical shape **(**Fig. 5**F**).

In the absence of light attenuators, the energy fluence at the wavelength of 750, 800 and 850 nm (i.e., optical absorption of deoxy-blood, GNR@Chit-*Iso4* and oxy-blood) was 30–35 mJ/cm^2^. In the presence of 0.2% IL the energy fluence dropped to 9 mJ/cm^2^ and further decreased with higher concentrations of IL (Fig. 5**G**). These findings indicate that the maximum PA signal from GNRs is obtained by irradiation with energy fluence of 9 mJ/cm^2^.

In consideration of using the GNRs in vivo, because of the presence of tissues surrounding the murine bladder, we considered to use light attenuators with lower amount of IL. Using a Monte Carlo simulation we estimated the light energy at 800 nm wavelength that reaches the murine bladder, considering a distance of 0.3 cm from the skin to the bottom of the bladder.

We considered the light energy that was measured for estimating the light fluence in Fig. 5**G**, and at a depth of 0.3 cm the energy value in the absence of light attenuators was estimated to be 5.3 mJ, while using the light attenuator made of 0.1% IL the energy value was 2.458 mJ (Fig. 5**H,** Supplementary Fig. S11).

### 3.8 Setup for the detection of the PA signal of GNR in the murine bladder

Light attenuators containing 0.1% IL were selected for imaging GNRs@Chit-*Iso4* instilled in the murine bladder. A “*dotted-like*” PA signal was clearly visible in the murine bladder instilled with GNRs@Chit-*Iso4* (3 nmol Au) but not with a 100 μl vehicle (saline solution) (Fig. 5**I**). The PA spectrum shape of these “*dotted-like*” structures was similar to that observed in vitro, i.e., with a peak of 810 nm (Fig. 5**J**). This result suggests that GNRs@Chit-*Iso4* can be easily detected in vivo without any significant changes in the PA properties of the nanoparticles.

We identified that 2.458 mJ represents the energy value that allows to obtain the maximum PA signal of GNRs in the murine bladder in the absence of reshaping.

### 3.9 GNRs settle to the bottom of the bladder

Time-course PAUS analysis of GNRs@Chit-Iso4 (3 nmol) at 0, 5, 10, 20 and 30 min after installation showed a progressive signal accumulation toward the bottom bladder (Fig. 5**K**). In particular, starting from 5 min after installation only ∼25% of PA signal could be detected in the upper half of the bladder, and most of the signal remained stacked in the lower half of the bladder (Fig. 5**L**), a condition that did not allow for the detection of a tumor that was located in the upper/lateral part of the bladder (Fig. 5**M**). Of note, no settlement of GNRs was observed upon storage at 37 °C up to 2 h, suggesting that GNRs maintain their colloidal properties outside of the bladder.

### 3.10 Low frequency US-assisted shaking keeps GNR@Chit-*Iso4* in suspension within the bladder

With the intention of providing a technological platform for the early diagnosis of cancer located everywhere in the bladder, we aimed to deliver a strategy for keeping GNRs in suspension in the bladder using low frequency US-assisted shaking.

**Fig. 6:**
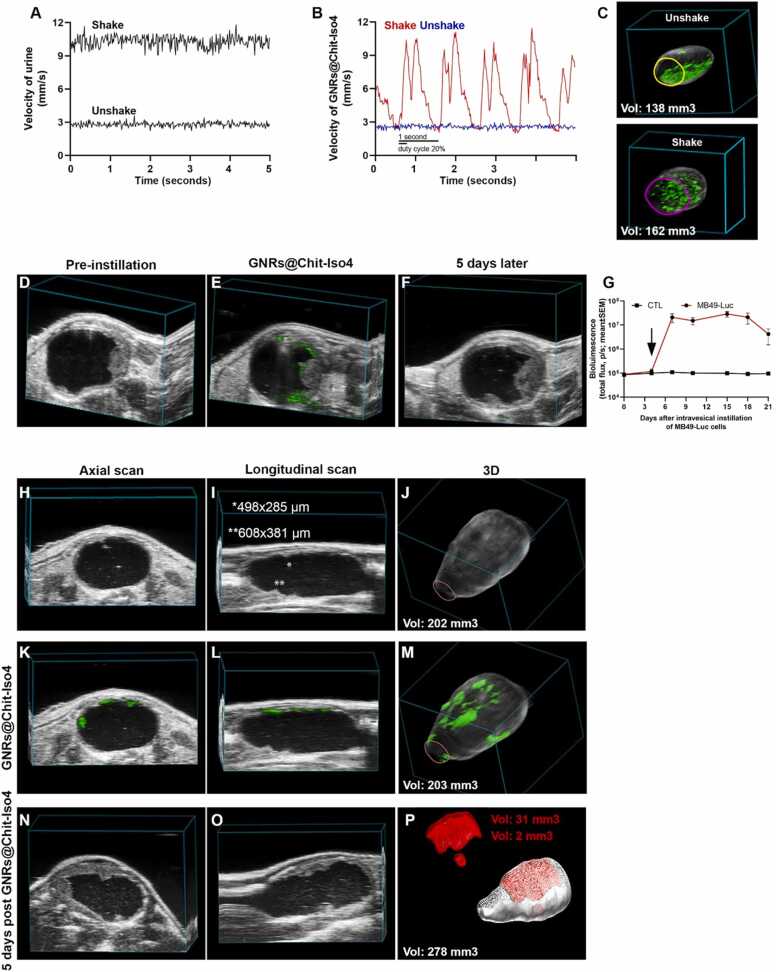
**Early diagnosis of bladder cancer. A**) Velocity of urine in unshake and shake condition (one representative experiment of four). **B**) Velocity of GNRs@Chit-*Iso4* (3 nmol Au) in unshake and shake condition (one representative experiment of four). **C**) 3D distribution of the GNRs@Chit-*Iso4* (3 nmol Au) PA signal in murine bladder in the unshake and shake condition (one representative experiment of four); the *green signal* corresponds to the PA signal of GNRs after unmixing the PA signal of melanin, deoxy- and oxy-genated blood and GNRs. **D**) Axial frame of a murine bladder with an US recognizable tumor nine days after intravesical instillation of MB49-Luc cells (one representative experiment of six); axial diameter of the bladder lumen = 4.8 mm. **E**) Axial frame of PAI of the same animal shown in panel D after the intravesical instillation of GNRs@Chit-*Iso4* (3 nmol Au), followed by 3 cycles of shaking, removal of the GNRs and 2 washes with saline solution (one representative experiment of six); axial diameter of the bladder lumen = 3.7 mm. **F**) PAI of the same animal used in panel D-E 5 days after the instillation of the GNRs@Chit-*Iso4* (one representative experiment of six); axial diameter of the bladder lumen = 4.2 mm. **G**) Time course bioluminescence of 12 animals without or with orthotopic bladder tumors derived from intravesical instillation of MB49-Luc cells (mean±SEM); the arrow indicates the day when the PAI was acquired to diagnosis the orthotopic bladder tumor in the experiment reported in panel H-M. Axial (**H**; diameter of bladder lumen= 5.5 mm) and longitudinal (**I**; diameter of bladder lumen = 11 mm) frames and 3D reconstruction (**J**) of US imaging of the murine bladder 4 days after the intravesical instillation of MB49-Luc cells (one representative experiment of four). Axial (**K**; diameter of bladder lumen = 6.5 mm), longitudinal (**L**; diameter of bladder lumen = 11 mm) and 3D distribution (**M**) of the GNRs@Chit-*Iso4* (3 nmol Au) PA signal in the murine bladder with tumor not detectable by US imaging and bioluminescence (day 4 after intravesical instillation of MB49-Luc cells) (one representative experiment of four). Axial (**N**; diameter of bladder lumen = 6.2 mm) and longitudinal (**O**; diameter of bladder lumen = 12 mm) frames, and 3D reconstruction (**P**) of US imaging of bladder and orthotopic tumor three days after tumor recognition by GNRs@Chit-*Iso4* (one representative experiment of four). Vol: Volume.

Low frequency US stimulation increased the velocity of urine from 2.9 ± 0.2 mm/sec to 10.4 ± 0.5 mm/sec (Fig. 6**A**) and of GNRs@Chit-*Iso4* from 2.6 ± 0.1 mm/sec to 11 ± 0.5 mm/sec (Fig. 6**B**). Furthermore, the velocity of urine remained constant (Fig. 6**A**) while GNRs showed a bimodal distribution according to the 1 s impulse and the fraction of period in which the signal was active (duty cycle 20%) (Fig. 6**B**). US-assisted shaking increased the velocity and volumetric flow rate of GNR@Chit-*Iso4* by at least 6-fold in all bladder regions, while maintaining the difference between different bladder areas (Supplementary Fig. S12). This strategy was sufficient to suspend the GNR@Chit-*Iso4* in the entire volume of the bladder (Fig. 6**C**).

### 3.11 Feasibility of the technological platform for the specific recognition of bladder cancer

We verified whether the PAI of US-assisted shaking of urine stable GNRs@Chit-*Iso4* could be exploited for the diagnosis of the well-established orthotopic α5β1^+^ bladder tumor (i.e., the murine MB49-Luc derived tumor) (Fig. 6**D**). To this aim, PAI was performed after the intravesical instillation of GNRs@Chit-*Iso4* or GNRs@Chit-Cys, followed by three cycles of US-assisted shaking and two intravesical washes to remove the unbound GNRs. We observed that the binding of GNR@Chit-*Iso4* was limited to the tumor cells, and not present in non-neoplastic tissue (Fig. 6**E**). Of note, five days later we confirmed the tumor growth only in the bladder region that was previously indicated by the GNR@Chit-*Iso4* (Fig. 6**F**). Accordingly, no binding of GNR@Chit-*Iso4* was observed in healthy mice or of GNR@Chit-Cys in tumor bearing mice (Supplementary Fig. S13, S14**)**, suggesting that the *Iso4* peptide was crucial for the efficient recognition of α5β1^+^ tumor cells. Interestingly, the binding of GNR@Chit-*Iso4* to MB49-Luc tumors was reduced by co-administration of an excess of free *Iso4* peptide, but not ARA peptide (Supplementary Fig. S15), confirming that the binding of GNR@Chit-*Iso4* to α5β1^+^ cells was mediated by the *Iso4* peptide.

### 3.12 Early diagnosis of bladder cancer

Next, we verified whether our technological platform could be exploited to detected small superficial tumors. To this aim, we analyzed the binding of GNR@Chit-Iso4 in a mouse bearing a small orthotopic tumor (i.e., 4 days after MB49-Luc cell implantation) which was undetectable by bioluminescence (Fig. 6**G**). The axial (Fig. 6**H**) and longitudinal US scans revealed only two potential neoplastic regions of about 500 µm in 1 single frame (Fig. 6**I**), but they were not appreciable in the 3D reconstruction (Fig. 6**J**). On the contrary, the PA signal of GNR@Chit-*Iso4* allowed for the visualization of several neoplastic areas in the axial and longitudinal scans and 3D reconstruction (Fig. 6**K-M**). This finding was next confirmed by US imaging three days later, showing the presence of a well-established tumor in the same areas previously recognized by GNR@Chit-*Iso4*, i.e., the upper and lower part of the bladder (Fig. 6**N-P**). These data suggest that our technological platform allows for the recognition of small and flat bladder cancers that are not detectable using bioluminescence or US imaging.

### 3.13 The technological platform does not induce bladder inflammation or disease spreading

**Fig. 7:**
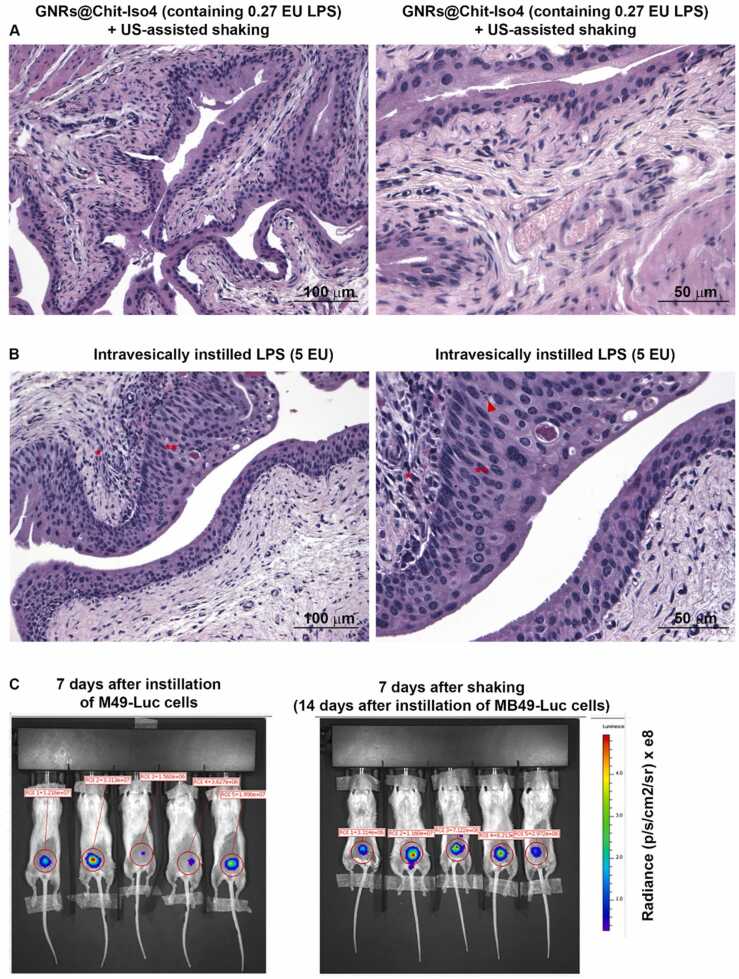
**Safety of the US assisted shaking of intravesical instilled GNRs@Chit-Iso4.** Hematoxylin/eosin staining of mice bladder seven days after intravesical administration of **A**) GNRs@Chit-Iso4 (30 nmol Au, equivalent to 27 mEU of LPS) or **B**) LPS (5 EU), followed by US-assisted shaking for 12 min and two intravesical washes. * ; inflammatory infiltrate in the lamina propria, * *; hyperplasia of the urothelium, arrowhead; apoptotic bodies. Low and high magnification are shown on the left and right panels, respectively. **C)** Bioluminescent imaging of mice at day 7 after intravesical instillation of MB49-Luc cells (left), and at day 7 after intravesical instillation of 3 nmol GNRs@Chit-Iso4 and US-assisted shaking.

The safety of our technological platform was then assessed at the local and distant level seven days after treatments. The histological analysis of healthy mice bladders instilled with GNR@Chit-*Iso4* (30 nmol Au), containing 27 mEU/ml of endotoxin showed no signs of inflammation or cell proliferation (Fig. 7**A**), while control mice intravesically instilled with 5 EU of lipopolysaccharides (LPS) showed strong inflammation characterized by the presence of an inflammatory infiltrate in the lamina propria, hyperplasia of the urothelium and apoptotic bodies (Fig. 7**B)**. Furthermore, whole-body in vivo bioluminescent imaging of orthotopic MB49-Luc tumor bearing-mice showed that the tumor remained confined to the bladder and did not spread (Fig. 7**C**), even after three cycles of US-assisted shaking.

## 4 Discussion

With the aim of providing a technological platform for the early diagnosis of small, flat shaped and aggressive bladder cancer lesions such as the bladder CIS, we developed a combination of strategies that allow for the detection of this tumor with an unprecedented sensibility. The results show that the combination of PAI with low frequency US-assisted shaking of intravesically instilled GNRs@Chit-*Iso4* in an orthotopic model of bladder cancer is capable of revealing the presence of lesions undetectable with US imaging and bioluminescence. Of note, with this technological platform we could detect neoplastic lesions smaller than a half millimeter, with a sensitivity that far exceeds that of the US and CT urography for bladder carcinoma [5].

Some of the clinical practices currently in use for the management of bladder cancer (i.e., bladder draining, incubation time for the intravesical instillation of drugs for diagnostic purposes or adjuvant treatment for NMIBC, such as 5-ALA and Hexviv® or Mytomycin C and BCG, and electromotive administration of Mytomycin C to increase drug distribution) were adapted to this new technological platform (called EDIT; Early DIagnosis of Tumor). This platform involves minimally invasive procedures, consisting of catheterization for bladder washing and the intravesical instillation of GNRs, the application of non-ionizing US for maintaining GNRs in suspension, and the use of pulsed laser light in the near-infrared biological window to obtain molecular imaging from targeted GNRs and 3D morphological reconstructions of the bladder.

As a contrast agent for diagnostic imaging we opted for the use of targeted GNRs, designed with a diameter of 10 nm and aspect ratio of 3.6 in order to have a peak light absorption at 808 nm, to leverage the optical window that allows for deeper tissue penetration [40], [41] and to overcome the different endogenous contrast molecules present in tissues [18]. We established the PA dynamic range of the above GNRs and identified the maximum fluence and energy of the pulsed laser light to obtain diagnostic imaging using targeted GNRs avoiding reshaping of the nanostructure.

The urinary bladder environment offers the possibility to exploit the intravesical instillation of GNRs, which is characterized by pro and cons compared to systemic delivery. Intravesical instillation allows for the avoidance of off target effects and off-target accumulations, such as the accumulation of gold in the liver, spleen, kidney, testis and brain, as has been observed in cases of systemic instillation [42], [43]. On the other hand, intravesical delivery of the treatment i) must content with urine, which contains a broad variety of byproducts from the metabolism of endogenous and exogenous substances [37], bacteria [38], [44], bacteria-derived mucus and floating urothelial cells, ii) is characterized by temporary retention, and iii) cannot exploit the enhanced permeability of the tumor vasculature and retention effects of the neoplastic vasculature to accumulate the intravenously injected GNRs in the neoplastic environment [45], [46], [47]. Moreover, as recently reported for the intravesical instillation of 100 µm silicon dioxide microparticles [48], the absence of a flow carrying the molecule allows for the quick deposition of GNRs on the bottom of the organ. Among the several pros of the PAI is the co-registration of US and PA signal, which allow to detect the organ of interest (US imaging) and the localization of the molecule under investigation (with PA property, i.e., molecular imaging). In the case of hollow organs as the bladder, the delivery of the nanoparticles does not take advantage of a flow carry the molecule, and their investigation with PA imaging subjects the particles to pressure exerted by ultrasounds that are used for ultrasound imaging. The most likely explanation why GNRs@Chit-Iso4, and microparticles, are settling to the bottom of the bladder during PAI, is that ultrasounds applied to the abdomen exert pressure on the urine contents toward the bottom of the bladder (Supplementary videos SV1 and SV2).

Supplementary material related to this article can be found online at doi:10.1016/j.pacs.2022.100400.

The following is the Supplementary material related to this article Video S1, Video S2.

Video S1

Video S2.

This phenomenon prevented the detection of tumors located at the top or lateral sides of the bladder. We solved this bias by applying a technique of non-invasive low-frequency US-assisted shaking of GNRs that increased the velocity and distribution of the nanoparticles in every region of the bladder, and prevented their precipitation to the lower half of the organ. We have established the parameters that permit to increase GNRs@Chit-*Iso4* distribution on the top and lateral sides of the bladder, ensuring that targeted GNRs come into contact with any urothelial area in which the tumor might be located. Compared to other protocols such as the electromotive drug administration used to improve bladder wall penetration of Mytomycin C [49], [50] or the use of a magnetic field to move silicon dioxide microparticles [48], US-assisted shaking from the abdomen is a safe, non-invasive protocol.

We identified integrin α5β1 as being expressed by the human bladder CIS and human bladder cancer cell lines, as well as expressed by the murine MB49-Luciferase (MB49-Luc) cell line and the MB49-Luc derived orthotopic syngeneic murine bladder cancer [51], but not by both the non-neoplastic human and murine urothelium. We investigated the use of the *Iso4* peptide that we have previously reported to selectively recognize the α5β1 integrin [25], and designed functionalized GNRs (GNRs@Chit-*Iso4*). The results of the present study also demonstrate that the peptide *Iso4* maintains its functional properties (i.e. α5β1 integrin recognition) after coupling to GNRs@Chit and in the presence of urine, and that upon coupling to GNRs it does not modify the PA spectra of the nanoparticles. This was observed with two batches of GNRs@Chit-*Iso4* loaded with 8.1 ± 10^6^ and 4.2 ± 10^6^ peptides/GNRs (Supplementary Table S7), or with aliquots stored at − 80 °C for > 1 year, suggesting that these nanoconstructs are reproducible and stable. We demonstrated the safety and feasibility of the EDIT platform to detect α5β1 integrin positive bladder tumors, such as the human bladder CIS.

The main clinical limitations of the EDIT approach for tumor imaging is related to the heterogeneity of tumor markers expressed among bladder cancer patients and the depth reached by the PAI. We found α5β1 expression in six out of eight (75%) specimens from patients with a diagnosis of bladder CIS, similar to what was previously reported for Cytokeratin 20 [52] in the bladder CIS or for EPCAM and uPAR in MIBC [53]; the combination of 2 or more targeting ligands, either coupled to the same or different GNRs, might be exploited to reach all bladder CIS. In this preclinical study a transducer with center frequency 40 MHz was used, which facilitates a spatial resolution of 40 µm and an imaging depth of 15 mm; to move to the clinic, lower frequency transducers will be investigated to achieve the imaging depth required for the human studies.

## 5 Conclusions

The feasibility of the EDIT platform in an animal model of bladder cancer identifies a new avenue for the early detection of bladder lesions < 1 mm in size in patients. Due to the heat releasing properties of the GNRs, this study also opens novel avenues for the early detection and therapy of bladder cancer [33], [54].

The results of this study show the feasibility of the early diagnosis of bladder cancer using the α5β1-targeted GNRs@Chit-*Iso4* conjugate as a photoacoustic contrast agent. Considering that the α5β1 integrin is also expressed by endometrial tumors [55], gastric cancer [56], breast cancer [57], and by the tumor neovasculature [58], [59], the photoacoustic imaging approach described here could be potentially exploited in the diagnosis of other solid neoplasia.

## Ethics approval and consent to participate

Human data collection followed the principles outlined in the Declaration of Helsinki. Patients signed an informed consent agreeing to supply their their own anonymous information and tissue specimens for future studies. The study was approved by the Institutional Review Board (Ethic Committee IRCCS Ospedale San Raffaele, Milan, Italy). All methods were carried out in accordance with the approved guidelines. All procedures and studies involving mice were approved by the Institutional Animal Care and Use Committees of San Raffaele Scientific Institute and performed according to the prescribed guidelines (IACUC, approval number 942).

## Funding

This study has received funding from the European Union’s Horizon 2020 research and innovation program under grant agreement No 801126 (https://cordis.europa.eu/project/id/801126). The funding source had no role in the design of this study, data interpretation, writing of the report.

## CRediT authorship contribution statement

Conception and design: MCF, FC, MA. Development of methodology: EA, SM, JJ, PG, MM, FC, MA. Acquisition of data: EA, MM, MiMa, IL, EL, ST, ASac, MN, RL, FP. Analysis and interpretation of data: all authors. Writing, review, and/or revision of the manuscript: AC, FM, ASal, FC, MCF, MA. Administrative, technical, or material support: SM, JJ, PG, MCF, FC, MA. Study supervision: MCF, FC and MA.

## Declaration of Competing Interest

The authors declare the following financial interests/personal relationships which may be considered as potential competing interests: Massimo Alfano reports financial support was provided by EU Framework Programme for Research and Innovation Future and Emerging Technologies.
