# Supplementary material for: Early diagnosis of bladder cancer by photoacoustic imaging of tumor-targeted gold nanorods
Source: Photoacoustics. 2022 Aug 30;28:100400. doi: 10.1016/j.pacs.2022.100400 (PMC9649962; doi:10.1016/j.pacs.2022.100400)
Supplement: Supplementary file 3 — Supplementary material [file mmc1.docx]

**SUPPLEMENTARY MATERIAL**

**Early diagnosis of bladder cancer by photoacoustic imaging of tumor-targeted gold nanorods**

Elisa Alchera, Matteo Monieri, Mirko Maturi, Irene Locatelli, Erica Locatelli, Silvia Tortorella, Angelina Sacchi, Angelo Corti, Manuela Nebuloni, Filippo Pederzoli, Francesco Montorsi, Andrea Salonia, Sandra Meyer, Jithin Jose, Pierangela Giustetto, Mauro Comes Franchini, Flavio Curnis, and Massimo Alfano.

**TABLE OF CONTENTS**

- **Methods; Synthesis of GNRs@CTAB and coating with chitosan (GNRs@Chit)**
- **Results; Characterization of Chitosan-coated GNRs (GNRs@Chit)**
- **Supplementary Tables**
- **Table S1. Panel of antibodies used for the characterization of human and mouse tissues by immunohistochemistry.**
- **Table S2. Panel of monoclonal antibodies used for the characterization of human and mouse cells for integrin expression on cell membrane by flow cytometry analysis.**
- **Table S3. Optical properties of skin, tissues surrounding the bladder and GNRs.**
- **Table S4. Quantification of α5 and β1 integrin expression on human primary urothelial cells and bladder carcinoma cell lines as determined by FACS analysis.**
- **Table S5. Quantification of α5 and β1 integrin expression on MB49-Luc murine bladder cancer cell as determined by FACS analysis.**
- **Table S6. Characterization of GNRs@CTAB, GNRs@Chit, GNRs@Chit-Cys and GNRs@Chit-iso4 by VIS-NIR, TEM, Zeta Potential, FAAS and gravimetric analysis.**
- **Table S7. Quantification of Iso4 loaded onto two different GNRs@Chit-Iso4 preparations as determined by aminoacid analysis content.**
- **Supplementary Figures**
- **Figure S1. Set up of materials and equipment used for *in vitro* visualization of GNRs by PAI.**
- **Figure S2. Experimental setup for US-assisted shaking of GNRs and US imaging acquisition in vivo.**
- **Figure S3. Expression of** α**5 and β1 integrins in Von Brunn’s nest and human bladder CIS.**
- **Figure S4. Expression of** α**5 and β1 integrins in human bladder according to tumor stage in one bladder from radical cystectomy.**
- **Figure S5. Synthesis and characterization of GNRs@CTAB and GNRs@Chit.**
- **Figure S6. TEM and SAED analysis of GNRs@CTAB.**
- **Figure S7. ^1^H-NMR spectra** **of chitosan, thiolated-chitosan and GNRs@Chit at 600 MHz.**
- **Figure S8. Characterization of GNR@Chit.**
- **Figure S9. TEM analysis of GNRs@Chit-*Iso4*.**
- **Figure S10. Representative** **HPLC chromatogram of the hydrolyzed products obtained from GNRs@Chit-Iso4 and GNRs@Chit-Cys.**
- **Figure S11. Monte Carlo model of light transport.**
- **Figure S12. Velocity and volumetric flow rate of GNRs@Chit-*Iso4* in the absence and presence of US-assisted shaking.**
- **Figure S13. GNRs@Chit-Iso4 does not bind to normal urothelium *in vivo*.**
- **Figure S14. GNRs@Chit does not bind to orthotopic MB49-Luc tumor.**
- **Figure S15. The binding of GNRs@Chit-Iso4 to neoplastic urothelium is specific.**
- **Supplementary Videos**
- **Video SV1. Settling of GNRs in the murine bladder during US imaging.**
- **Video SV2. US-assisted shaking improves the distribution of GNRs in the bladder.**
- **Supplementary References**

**MATERIALS and METHODS**

**Synthesis of GNRs@CTAB with maximal absorption at 800 nm.** Cetyltrimethylammonium bromide (CTAB)-coated GNRs (GNRs@CTAB) with a maximum absorption of 800 nm, were prepared according to a previously published procedure with minor modifications [1] and scaled to a volume of ~2 litres. The initial solution for the growth of GNRs was prepared by dissolving CTAB (31.14 g, 85.4 mmol) and sodium oleate (4.28 g, 14.0 mmol) in 1.8 L of warm water (~ 50°C) in a thermostatically controlled 2-L jacketed reactor, equipped with mechanical stirring. When the solution reached 30°C, 810 µL of AgNO_3_ solution (0.4 M in ultrapure water) were added and the mixture that was then incubated for 15 min without stirring. Then, under continuous stirring (700 rpm), 8.652 mL of HAuCl_4_ solution (0.1 M in ultrapure water) were added. Au(III) was reduced to Au(I) using sodium oleate for 90 min, after which 3.56 mL of hydrochloric acid (37 %) and 3.6 mL of ascorbic acid (0.079 M) were added to adjust the pH to 1.0 and to ensure the complete reduction of the gold precursor. Separately, a seed solution was prepared by dissolving 364 mg (1.0 mmol) of CTAB in 10 mL of warm water in a 50 mL round-bottomed flask. After cooling down to room temperature 25 µL of HAuCl_4_ solution (0.1 M) were added while stirring. The seeds were formed by quickly injecting 600 µL of ice-cold sodium borohydride (0.01 M) into the solution, causing the colour of the solution to change from yellow to brown indicating the formation of ultra-small gold seeds. Finally, after ageing the seed solution for 30 minutes at room temperature, 690 µL of it were added to the growth solution, which was vigorously stirred for 30 s then left undisturbed overnight at 30°C to allow for GNR growth. Purification of GNRs@CTAB was performed by i) centrifugation in 50-mL Falcon tubes (6,000 rpm for 100 min), ii) removal of 40 mL of the supernatant and iii) re-dispersion in 40 mL of ultrapure water, and the process was repeated 3 times. The final product was then collected in 200 ml of ultrapure water. The gold concentration in GNRs@CTAB was 2.09 mM, as determined by atomic absorption spectroscopy.

**Coating of GNRs with chitosan (GNRs@Chit).** Medical grade chitosan (500 mg, 3.1 mmol) was dissolved in 50 mL of 1 vol. % acetic acid and mixed with 0.5 mL (7.2 mmol) of thioglycolic acid under moderate stirring. N-(3-dimethylaminopropyl)-N′-ethylcarbodiimide hydrochloride (500 mg, 2.6 mmol) was then added to activate the carboxylic group of thioglycolic acid and to promote the coupling to the amino groups of chitosan. The reaction was left to incubate for 6 h at room temperature under continuous stirring. The product was then dialyzed using a 3.5 kDa cut-off dialysis tube overnight against ultrapure water and the resulting thiolated-chitosan was diluted to 500 mL with water. At this point, 30 mL of GNRs@CTAB were added dropwise under mild stirring and the resulting solution was incubated (48 h, room temperature) to allow for the coupling of thiolated-chitosan and GNRs. The product was subsequently concentrated using an Amicon® stirred cell equipped with PES membranes (100 kDa cut-off, using 4 bar nitrogen pressure) to remove CTAB, and the final product (60 mL), called GNRs@Chit, was then stored at +4 °C until the subsequent step.

**RESULTS; CHARACTERIZATION OF CHITOSAN-COATED GNRs (GNRs@CHIT)**

GNRs were designed for peak absorption in the NIR-I biological window, centred around 800 nm. The synthesis of GNRs@CTAB was performed in a 2-L jacketed reactor (**Supplementary Figure S5A**), which produced 200 mL of 2.09 mM GNRs@CTAB (Yield = 49%), determined by FAAS, and a total dry matter of 0.8 mg/mL. These findings indicate that the gold content was approximately 60% of the nanosystem mass, in agreement with the fact that the CTAB completely covers the gold core and an excess of surfactant in solution is required to keep the nanosystem stable. Also the surface zeta potential + 29.8 mV was in agreement with the model of CTAB-capped gold nanosystems [2]. A primary evaluation of the geometrical anisotropy of GNRs@CTAB showed the presence of two distinct absorption bands (**Supplementary Figure S5B**), revealing that GNRs were obtained at a longitudinal-LSPR peak of 798 nm, while the energy-dispersive X-ray spectroscopy (EDX) spectrum revealed gold as the principal component of the identified structures (**Supplementary Figure S5C**). We then confirmed that the replacement of CTAB with chitosan did not modify the shape, morphology (**Supplementary Figure S5D**), or optical properties of the GNRs (**Supplementary Figure S5E**). This result is coherent with the previously observed optical behaviour of the systems.

Transmission electron microscopy (TEM) (**Supplementary Figure S6A**) showed the presence of cylindrical gold nanostructures with width (25.7 ± 2.0 nm) and length (88.2 ± 6.4 nm) corresponding to an aspect ratio of 3.43 ± 0.52, compatible with the NIR optical behaviour of the nanosystem[1], while the selected area electron diffraction (SAED) allowed for cross-validation of the expected crystalline structure of the particles (**Supplementary Figure S6B and S6C**). Overall, TEM experiments confirm the effective synthesis of monocrystalline GNRs with the appropriate aspect ratio. In this study, GNRs were coated with chitosan, a material widely used in biomedical applications owing to its proven biocompatibility with human tissues, and also used in the field of nanoparticles due to its capacity to stabilize GNRs during the synthesis process, not including any toxic reagent. Moreover, the abundance of amino groups in chitosan permits the functionalization with amine reactive linkers that allows the conjugation with several drugs, antibodies or peptides [3, 4]. GNRs@CTAB were capped with chitosan using a thiolated-chitosan conjugate. The insertion of a thiol group on chitosan monomers was performed through carbodiimide-assisted coupling between amino groups of chitosan and the carboxylic acid moiety of thioglycolic acid, and the effective modification of chitosan with thiol groups was confirmed by the mean of ^1^H-NMR (**Supplementary Figure S7A and S7B**).

The replacement of CTAB with Chitosan-SH on GNRs@CTAB was performed by dropping the GNRs-CTAB solution into a solution of Chitosan-SH diluted in water, and the complete removal of CTAB from GNRs@Chit was assessed by ^1^H-NMR (**Supplementary Figure S7C**) and by examining the surface zeta potential of purified GNRs that ranged from + 35 mV to + 45 mV. Compared to the GNRs@CTAB, the increased zeta potential suggests the replacement of CTAB being that the residual non-functionalized amino groups of chitosan preserve their cationic nature in water.

Next, the freeze-dried product underwent thermogravimetric analysis (TGA) to determine its thermal behaviour (**Supplementary Figure S8A**) revealing a total inorganic content of 2.1%. The rheology of GNRs@Chit 1 mM in water was evaluated by measuring the viscosity and shear stress, varying the applied shear rate (**Supplementary Figure S8B, S8C**), showing shear-thinning behaviour with higher applied stress. Moreover, the viscosity of the solution was evaluated by varying the temperature from +4°C to +40°C (**Supplementary Figure S8D**), revealing a constant decrease of the viscosity with increasing temperature. These findings indicate that GNRs@Chit were made of 2% Au and 98% chitosan, and that this solution is suitable for intravesical instillation.

**Supplementary Tables**

**Table S1. Panel of antibodies used for the characterization of human and mouse tissues by immunohistochemistry.**

| **Antigen** | **Host** | **Antibody**  **clone** | **Reactivity** ^b^**^)^** | **Dilution** | **Antibody Isotype** | **Vendor (catalog)** |
| --- | --- | --- | --- | --- | --- | --- |
| α5 | Rabbit | EPR7854 ^a)^ | h/m/r | 1/100 | IgG | Abcam  (ab15031) |
| β1 | Rabbit | EPR16895 ^a)^ | h/m/r | 1/1000 | IgG | Abcam (ab179471) |
| None | Rabbit | Polyclonal | None | - | IgGs ^c)^ | Primm |

a) Monoclonal antibody. b) *h/m/r*, human, mouse and rat (according to *technical data sheet*). c) Isotype-matched negative control antibodies were prepared through Protein-A Sepharose purification.

**Table S2. Panel of monoclonal antibodies used for the characterization of human and mouse cells for integrin expression on cell membrane by flow cytometry analysis.**

| Antigen | Host | Antibody  (clone) | Reactivity ^b)^ | Antibody Isotype | Vendor  (catalog) |
| --- | --- | --- | --- | --- | --- |
| α5 | Mouse | P1D6 | h | IgG_3_ | Merk  (MAB-1956z) |
| β1 | Mouse | P5D2 | h | IgG_1_ | Merck  (MAB1959z) |
| None ^a)^ | Mouse | MOPC-21 | None | IgG | Sigma  (M 5284) |
| α5 | Hamster | HMα5-1 | m, r | IgG | Biolegend  (103902) |
| β1 | Hamster | HMβ1-1 | m, h | IgG | Biolegend  (142602) |
| None ^a)^ | Hamster | None | None | IgG | eBioscience  (14-4888-85) |

a) Isotype-matched negative control antibody. b) *h/m/r,* human, mouse and rat (according to *Technical Data Sheet*).

**Table S3. Optical properties of skin, tissues surrounding the bladder and GNRs.**

|  | Skin line^1^ | Standard tissue^1^ | GNRs^1^ | Urine^1^ |
| --- | --- | --- | --- | --- |
| Absorption coefficient (μa) [1/mm] | 0.4275 | 0.1 | 42.93 | 0.002 |
| Scattering coefficient (μs) | 25 | 10 | 0.521 | 1 |
| Scattering anisotropic factor (*g*) | 0.9 | 0.9 | 0.9 | 1 |
| Refractive index (*n*) | 1.33 | 1.44 | 1.36 | 1.333 |

For the urine, the absorption coefficient, scattering coefficient, and refractive index were assumed to be the same as those of water; the scattering anisotropic factor was chosen to be 1, implying that photons propagate inside the urine but do not undergo scattering. Values for the skin, standard tissue and GNRs from recent publication [5].

**Table S4. Quantification of α5 and β1 integrin expression on human primary urothelial cells and bladder carcinoma cell lines as determined by FACS analysis.**

|  |  | Primary Urothelial Cells | | | | Bladder carcinoma cell lines | | | | | | | |
| --- | --- | --- | --- | --- | --- | --- | --- | --- | --- | --- | --- | --- | --- |
| Antigen | mAb clone | PCS-420-010 ^a^ | | HBLAK ^b^ | | RT4 ^b^  *(T1, G1-2) ^c^* | | RT112 ^a^  *(Ta, G2) ^c^* | | 5637 ^a^  *(G2) ^c^* | | HT1376 ^a^  *(>T2; G3) ^c^* | |
|  |  | *n* ^d^ | Fold ^e^ | *n* | Fold | *n* | Fold | *n* | Fold | *n* | Fold | *n* | Fold |
| α5 | P1D6 | 1 | 1.5 | 1 | 2.1 | 2 | 6.0 ± 3.8 | 3 | 5.8 ± 2.2 | 2 | 1.9 ± 0.1 | 3 | 4.1 ± 0.8 |
| β1 | P5D2 | 1 | 1.2 | 1 | 0.8 | 2 | 18.6 ± 4.8 | 3 | 38.7 ± 19.9 | 3 | 40.5 ± 16.6 | 3 | 11.9 ± 2.9 |

Flow cytometry analysis of human primary urothelial cells (PCS-420-010) and human bladder 5637 cancer cell line stained using the indicated anti-integrin antibodies (5 μg/ml), followed by a goat anti-mouse Alexa Fluor 488-labeled secondary antibodies (5 μg/ml).

a) Derived from a female donor. b) Derived from a male donor. c) Stage and tumor grade of cancer cells. d) *n*, number of independent experiments each in duplicate. e) *Fold*, ratio of the median fluorescence intensity of a given anti-integrin antibody over the median fluorescence intensity of an isotype control matched antibody, mean ± SEM.

The gating strategy is reported in the panels following the supplementary Table S2.

**Table S5. Quantification of α5 and β1 integrin expression on MB49-Luc murine bladder cancer cell as determined by FACS analysis.**

| Antigen | mAb clone | MB49-Luc | |
| --- | --- | --- | --- |
|  |  | *n ^a^* | Fold ^b^ |
| α5 | HMα5-1 | 2 | 3.79 ± 0.18 |
| β1 | HMβ1-1 | 2 | 8.02 ± 1.04 |

Flow cytometry analysis of MB49-Luc bladder cancer cell stained with the indicated rabbit anti-integrin antibodies (5 μg/ml), followed by a goat anti-hamster Alexa Fluor 488-labeled secondary antibodies (5 μg/ml).

a) *n*, number of independent experiments each in duplicate. b) *Fold*, ratio of the mean fluorescence intensity of a given anti-integrin antibody over the mean fluorescence of an isotype control matched antibody, mean ± SEM.

**
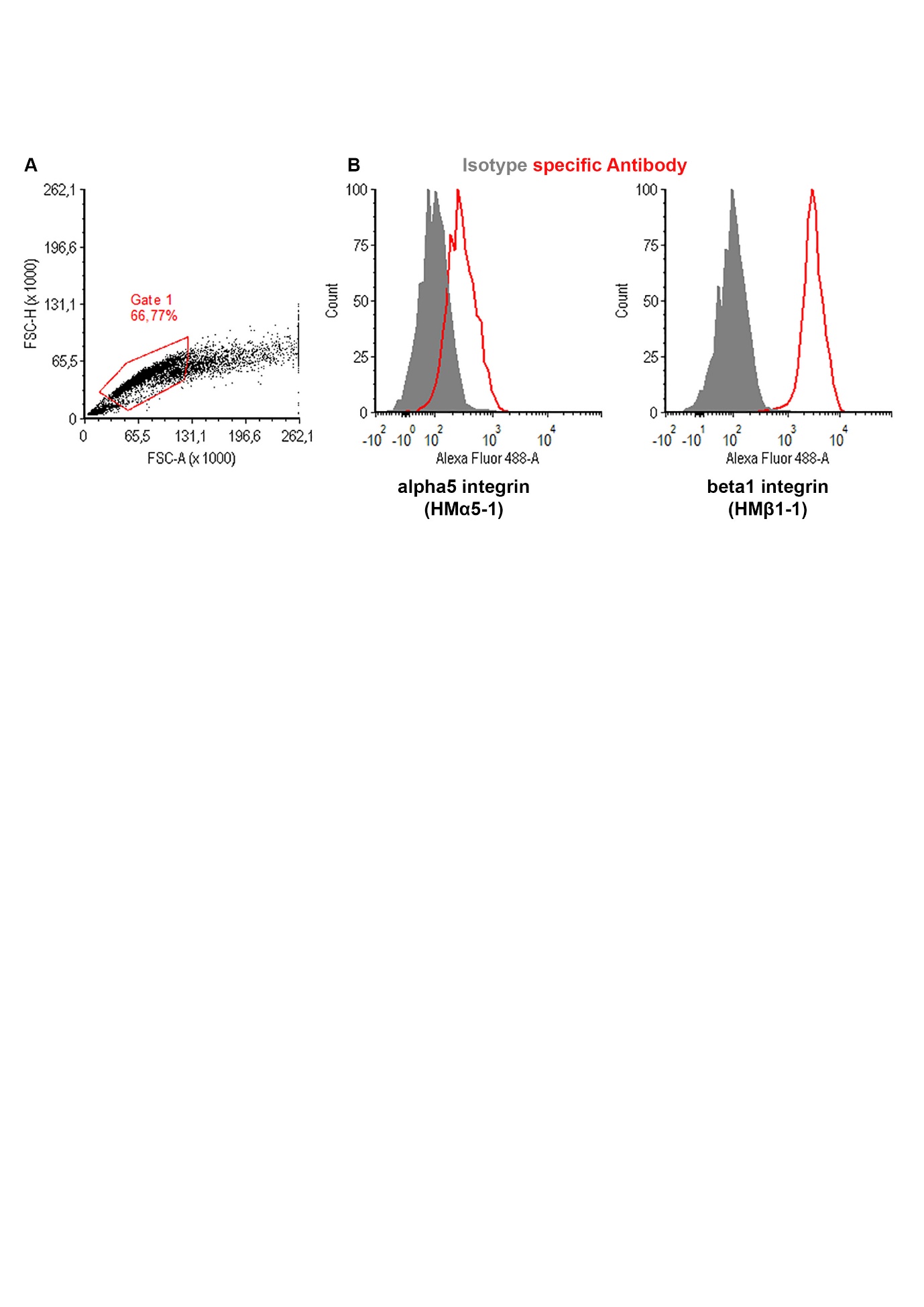
**The gating strategy is reported in the following panels; **A**) debries, duplets and multiplets were excluded based on physical parameters; **B**) ten thousand cells were acquired from gate 1 corresponding to singlets.

**Table S6. Characterization of GNRs@CTAB, GNRs@Chit, GNRs@Chit-Cys and GNRs@Chit-iso4 by VIS-NIR, TEM, Zeta Potential, FAAS and gravimetric analysis.**

| **Type of analysis** | **GNRs** | | | | | | |
| --- | --- | --- | --- | --- | --- | --- | --- |
|  | **GNRs@CTAB** |  | **GNRs@Chit** |  | **GNRs@Chit-Cys ^a^** |  | **GNRs@Chit-*Iso4* ^a^** |
| *VIS-NIR* |  |  |  |  |  |  |  |
| λ_max_ (nm) ^b^ | 798 |  | 800 |  | 802 802 | | |
|  |  |  |  |  |  |  |  |
| *TEM* |  |  |  |  |  |  |  |
| GNRs length (nm) | - |  | 88.2 ± 6.4c |  | 90.2 ± 7.2d 90.2 ± 7.2d | | |
| GNRs width (nm) | - |  | 25.7 ± 2.0c |  | 24.9 ± 2.6d 24.9 ± 2.6d | | |
| Aspect Ratio ^e^ | - |  | 3.4 ± 0.5 |  | 3.6 ± 0.6 3.6 ± 0.6 | | |
|  |  |  |  |  |  |  |  |
| *Zeta potential* (mV) | + 29.8 mV |  | + 40 mV |  | + 10.6±0.4 + 10.3±0.4 | | |
|  |  |  |  |  |  |  |  |
| *FAAS ^f^* |  |  |  |  |  |  |  |
| Au (%) ^g^ | - |  | - |  | 2.01 |  | 1.97 |
| Au (mM) | - |  | - |  | 1.00 |  | 1.00 |
| GNRs/ml ^h^ | - |  | - |  | ~ 2x10^11^ |  | ~ 2x10^11^ |
| *Gravimetric analysis* |  |  |  |  |  |  |  |
| Dry matter (mg) ^i^ | - |  | - |  | 4.90 |  | 5.00 |
|  |  |  |  |  |  |  |  |

a) After resuspension of a lyophilized vial with 0.5 ml of water. b) λ_max_: wavelength of max absorbance. c) n = 150 by TEM. d) n = 300 by TEM. e) Calculated as the length/width ratio. f) After redispersion of the content of one vial in 0.5 mL of water. g) Calculated as the ponderal ratio between gold content and total dry mass per vial. h) Calculated approximating the GNRs as half-sphere-capped cylinders. i) Weight of the dry content of each vial.

**Table S7. Quantification of Iso4 loaded onto two different GNRs@Chit-Iso4 preparations as determined by aminoacid analysis content^a^.**

| **Gold nanorods** | **Batch**  **code** | **Peptide added (mg)^b^** | **Peptide found (mg)** | **Coupling**  **Efficiency (%)** | **N° of peptides**  **/GNR** | **Peptide density**  **(N° of peptides/μm^2^)^d^** |
| --- | --- | --- | --- | --- | --- | --- |
| GNRs@Chit-**Iso4** | #A | 4.74 | 4.3 ± 0.1  *(2) ^c^* | 91 ± 2 | 8.1 (±0.4) x10^6^ | 9.0 x10^8^ |
| GNRs@Chit-**Iso4** | #B | 21 | 14.6 ± 0.5  *(2) ^c^* | 69 ± 2 | 4.2 (±0.1) x10^6^ | 4.7 x10^8^ |

a) Lyophilized GNRs@Chit-Iso4 were resuspended in water (0.5 mM of Au, 1 x10^11^ NPs/ml) at 5 mg/ml (based on their dry-matter content) and subjected to acidic hydrolysis (20 hours at 110°C, in 6 M hydrochloric acid, 0.1% phenol, 0.1% thioglycolic acid under reduced pressure in an atmosphere nitrogen). The amino acid content in the hydrolyzed products was then quantified using ion exchange chromatography and post-column derivatization with ninhydrin. The hydrolyzed product obtained from GNRs@Chit-Cys was used to establish the background (**Supplementary Figure S8**). b) By Ellman’s quantification. c) Number of independent quantifications. d) The density of peptides per μm^2^ of GNR was calculated considering each GNR as cylinders capped with two half-spheres and a surface of 0.009 μm^2^. Data shown as mean±SE.

**Supplementary Figures**

**
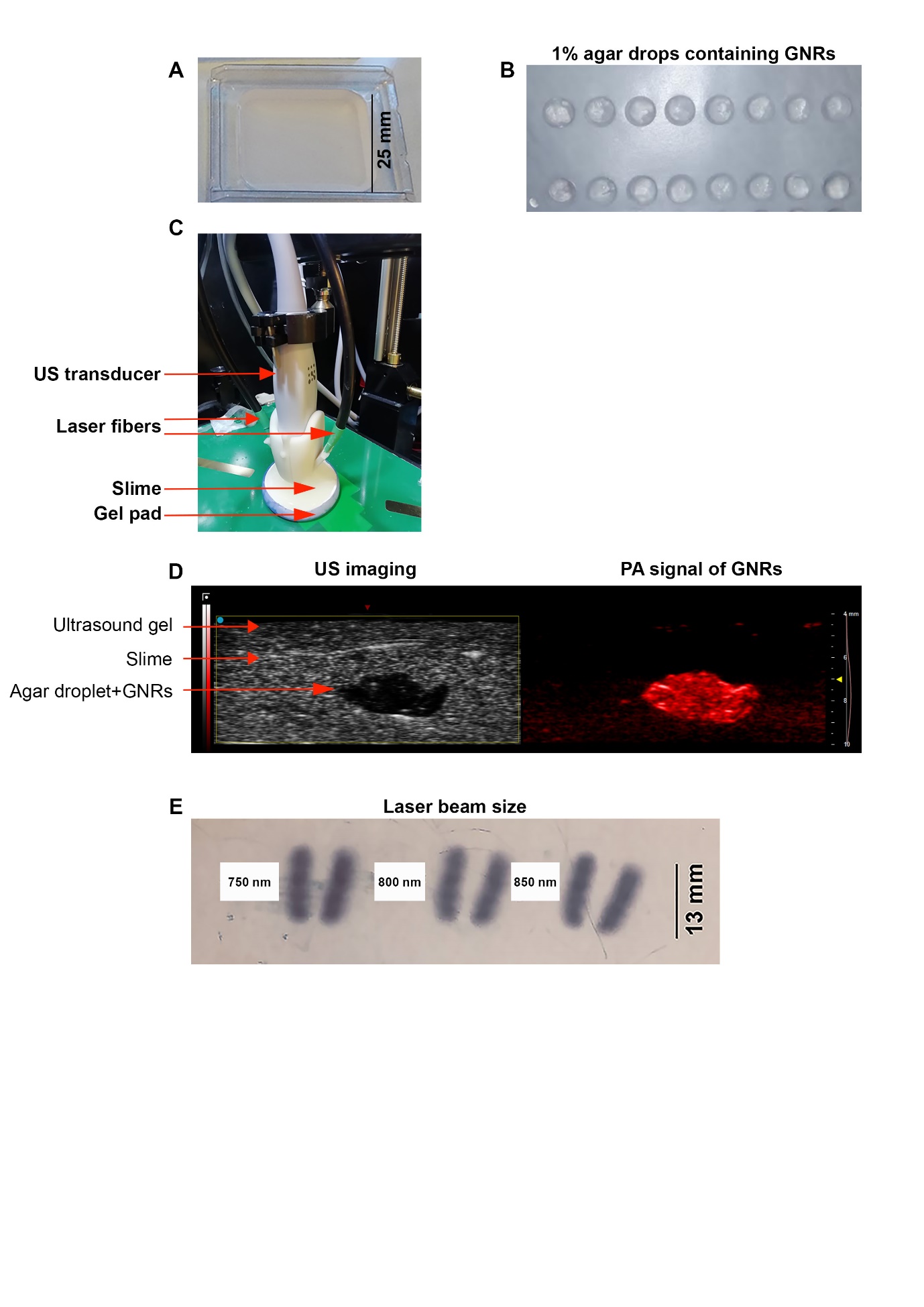
**

**Figure S1. Set up of materials and equipment used for *in vitro* visualization of GNRs by PAI.**

**A**) A representative light attenuator made of 1% agar and IL within dispomold cassettes.

**B**) Example of 1% agar drops containing GNRs.

**C**) Instrument set up for PAI of agar drop containing GNRs.

**D**) US imaging and PA signal of one representative agar drop containing GNRs@Chit-Iso4 (15 nmol) embedded into the slime and acquired using the light attenuator made of 1% agar and 0.6% IL; the echogenic signal (gray) is generated by the slime in which the agar drop is embedded.

**E**) Laser beam on a photographic paper placed at 8 mm from the laser fibers and exposed to the indicated wavelengths for 5 sec. The length and width of each spot was 13 and 3 mm, respectively.

**
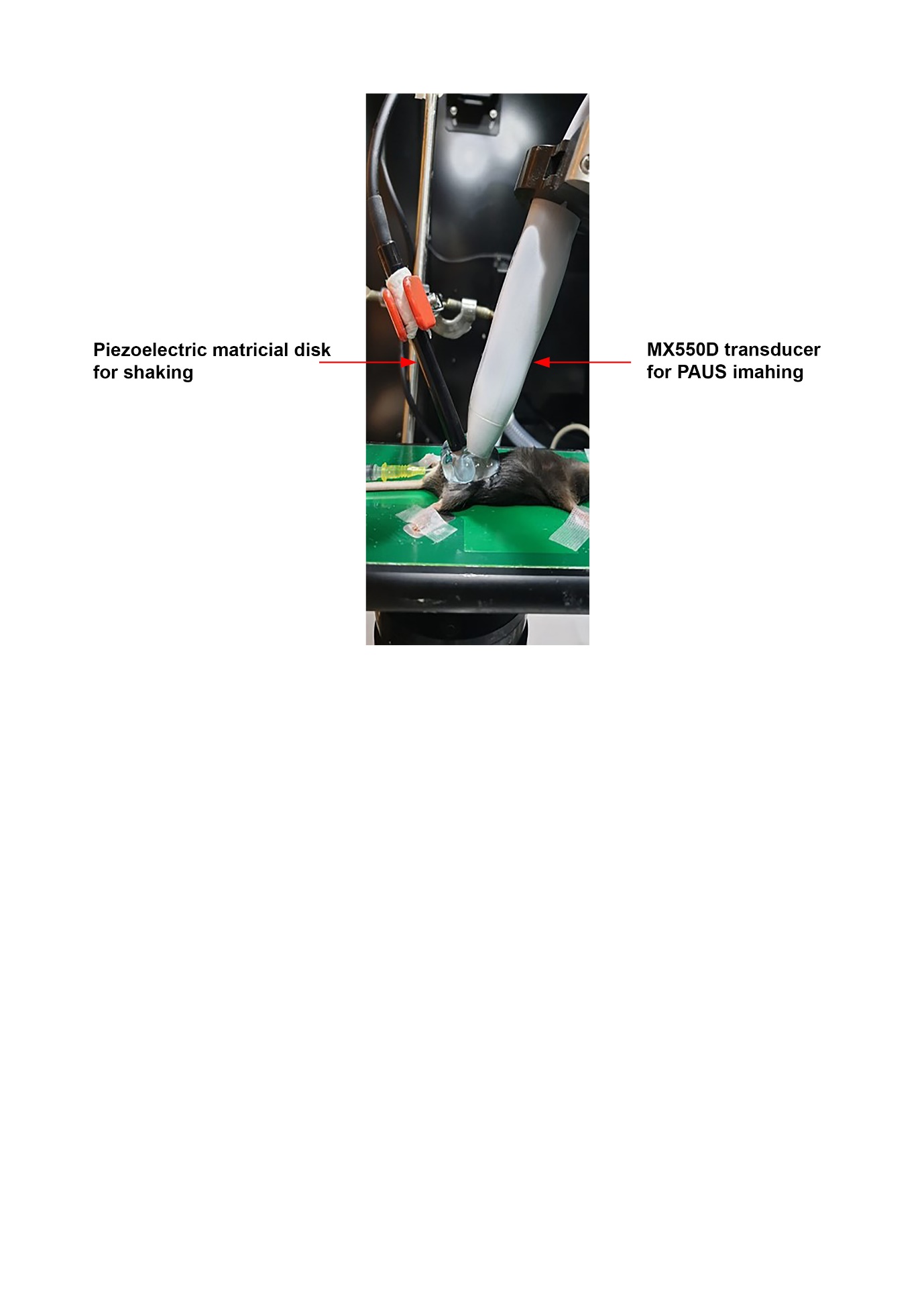
**

**Figure S2. Experimental setup for ultrasound-assisted shaking of GNRs and US imaging acquisition in vivo.**

The picture shows a mouse positioned on the dorsal side and restrained on the heating board with the piezoelectric matrix array transducer for shaking and the MX550D transducer placed on the abdomen and positioned each other with an angle of 45 degrees. The in-place intravesical catheter is also shown.

**
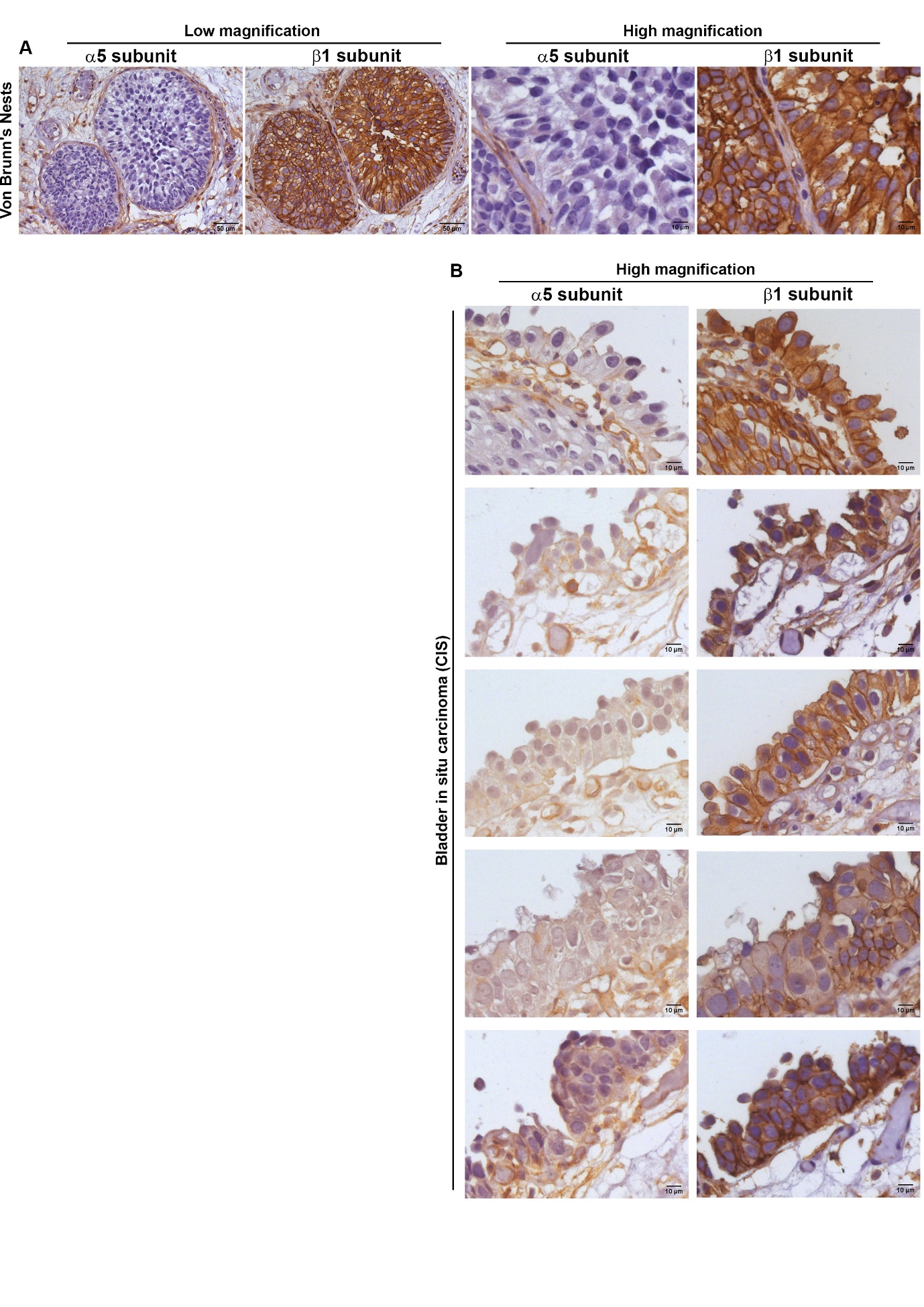
Figure S3. Expression of α5 and β1 integrins in Von Brunn’s nest and human bladder CIS.**

**A)** One representative immunohistochemistry photomicrographs of two tested human bladder sections of the normal urothelium present in the Von Brunn’s nest.

**B**) Five human bladder CIS with positive stain for α5 integrin at the membrane level, out of six CIS tested. All tissues were from TURB.

**
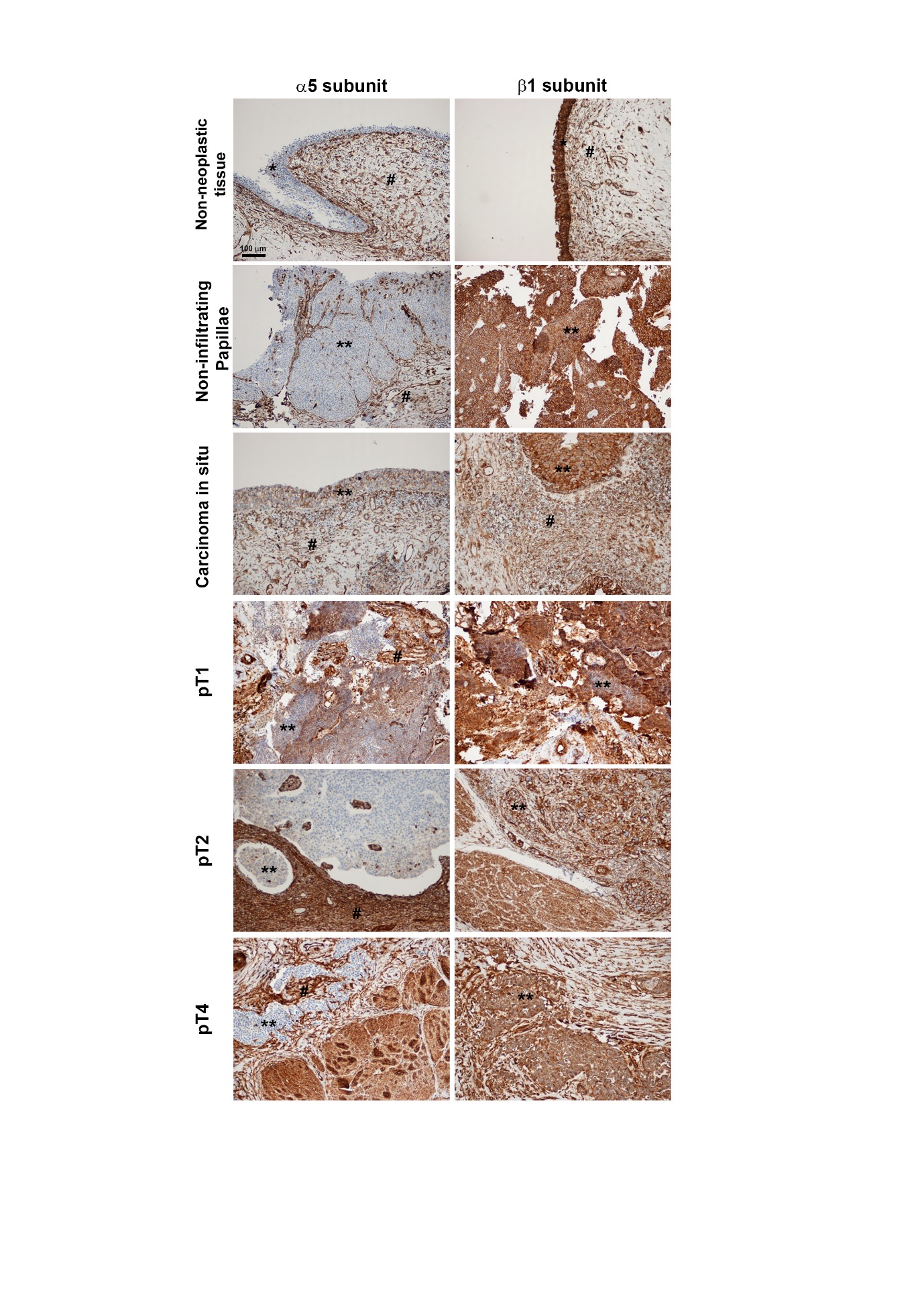
Figure S4. Expression of α5 and β1 integrins in human bladder according to tumor stage in one bladder from radical cystectomy.**

Representative immunohistochemistry photomicrographs of human bladder sections of one cancer patient subjected to radical cystectomy with paired non-neoplastic urothelium, NMIBC (pTa and Cis) and MIBC (pT2-pT4) and immunostained with the indicated anti-integrins antibodies. *non-neoplastic urothelium; #; stroma, **tumor tissue. Scale bar: 100 µm.

**
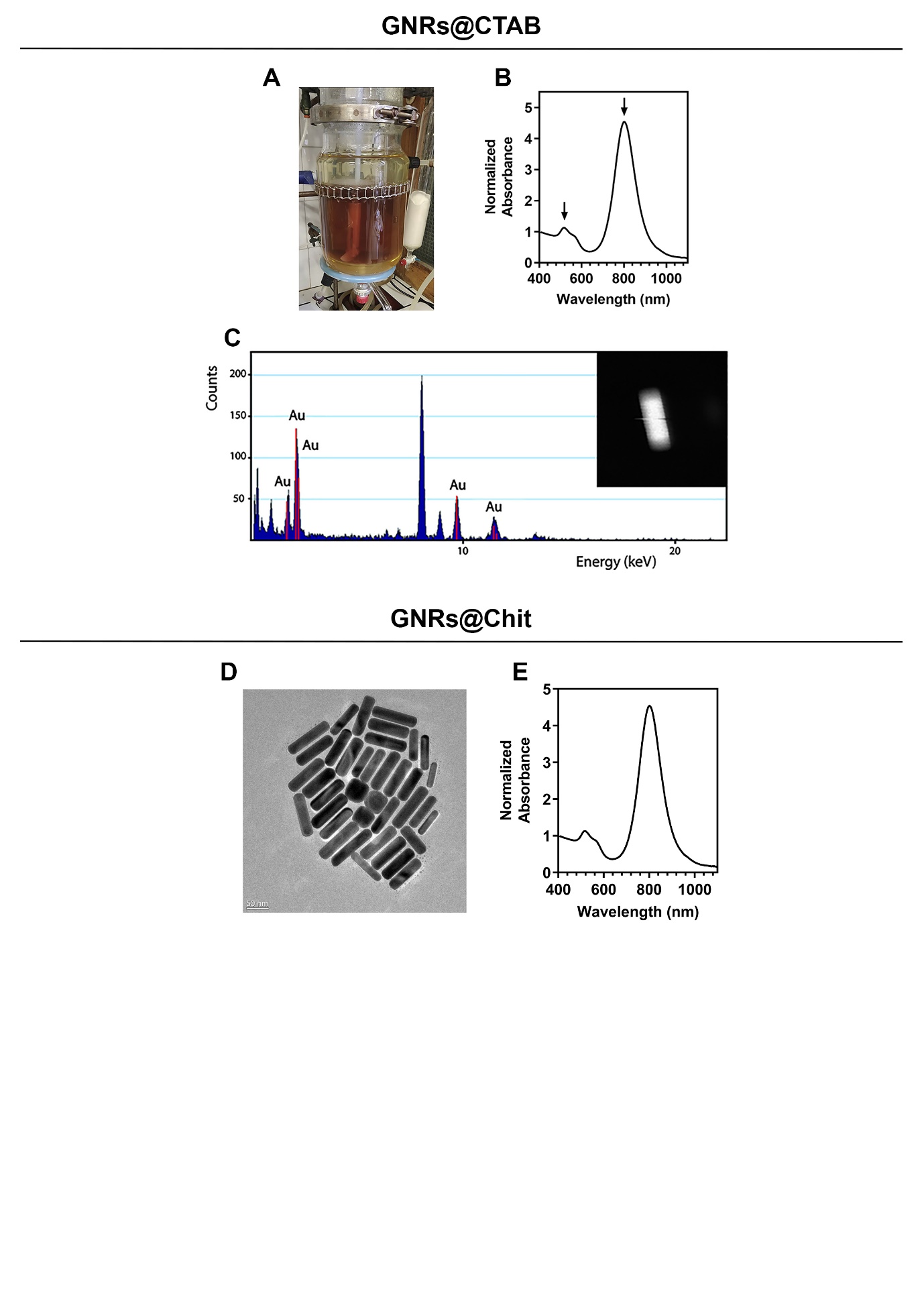
**

**Figure S5. Synthesis and characterization of GNRs@CTAB and GNRs@Chit**

**A)** Two liter jacket reactor equipped with mechanical stirrer for the large scale synthesis of GNRs.

**B**) VIS-NIR spectra of GNRs@CTAB showing the presence of two distinct absorption bands representative of the transversal (left) and longitudinal (right) surface plasmonic resonance.

**C)** Quantification of gold in GNRs@CTAB by EDX analysis.

**D**) Shape of GNRs@Chit by TEM analysis (scale bar = 50 nm).

**E)** VIS-NIR spectra of GNRs@Chit.

**
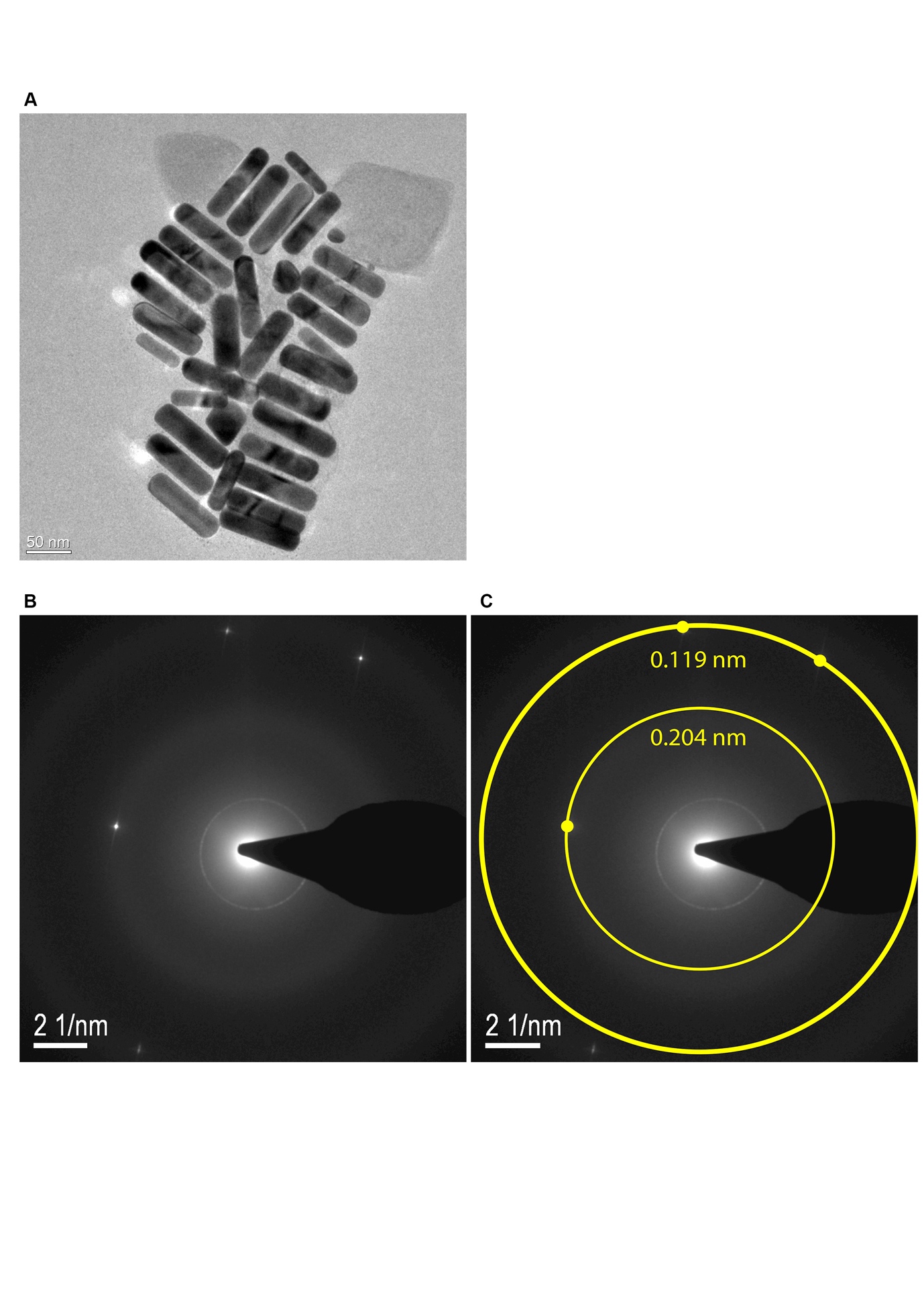
Figure S6. TEM and SAED analysis of GNRs@CTAB.**

**A**) Representative TEM image of GNRs@CTAB.

**B**) and **C**) selected area electron diffraction of a single GNR, revealing two distinct reflections assigned to the [200] and [222] sets of crystal planes of Au face-centered cubic structure. Two diffraction spots were present: by calculating the corresponding interplanar distances in GNRs electron diffraction, of 2.04 and 1.19 Ǻ have been determined. These correspond to distances between specific planes in gold FCC crystal structure (space group Fm3m), the [200] and [222] sets of planes, respectively.

**
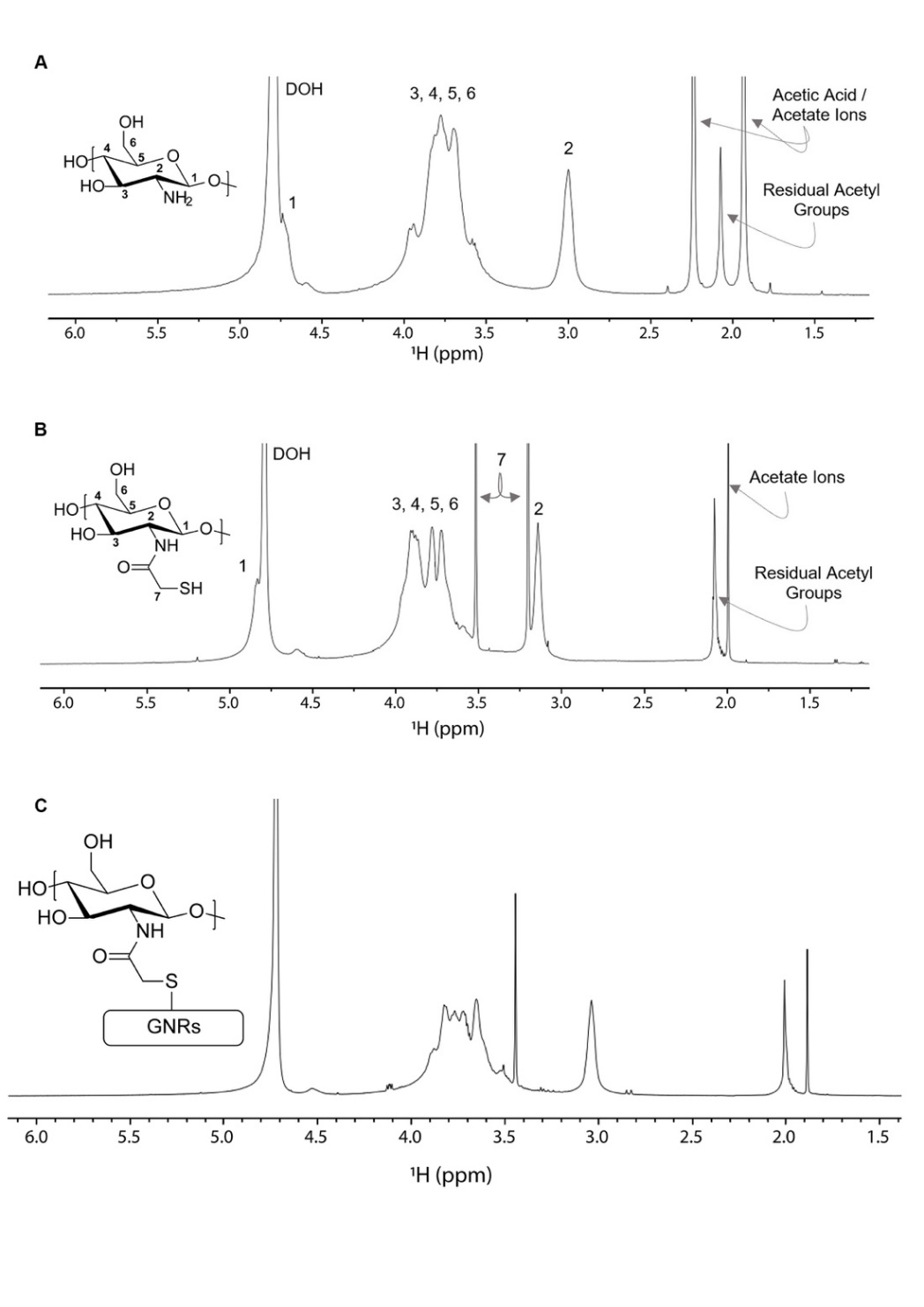
Figure S7. ^1^H-NMR spectra** **of chitosan, thiolated-chitosan and GNRs@Chit at 600 MHz.**

**A)** ^1^H-NMR (600 MHz, CH_3_COOH 1% in D_2_O) of chitosan.

**B**) ^1^H-NMR (600 MHz, D_2_O) of thiolated-chitosan. Full assignment of spectral features was performed by evaluating peak positions and intensities. The peaks corresponding to bound thioglycolic residues (7) are located at 3.2 and 3.5 ppm. By comparison, the obtained spectrum with the ones reported in the literature, the unambiguous assignment of peaks was possible [6]. After conjugation with thioglycolic acid, sharp singlet peaks appear at 3.20 and 3.50 ppm. This has been related to the CH_2_ residue of the thioglycolic moiety attached to the chitosan amino group, which splits upon partial deprotonation of the thiol group in a neutral aqueous environment.

**C**) ^1^H-NMR spectrum of GNRs@Chit (600 MHz, D_2_O). The obtained spectrum resembles all the features of the chitosan spectrum. The absence of signals coming from CTAB allows to state the success of the ligand exchange reaction. Moreover, it is clearly noticeable that the sharp peak corresponding to the CH_2_ on thioglycolic moieties does not display splitting due to thiol deprotonation equilibrium. This can be directly connected with the efficient attachment of chitosan on GNRs surface since the thiol group is now bound to gold atoms and protonation equilibrium is not allowed.

**
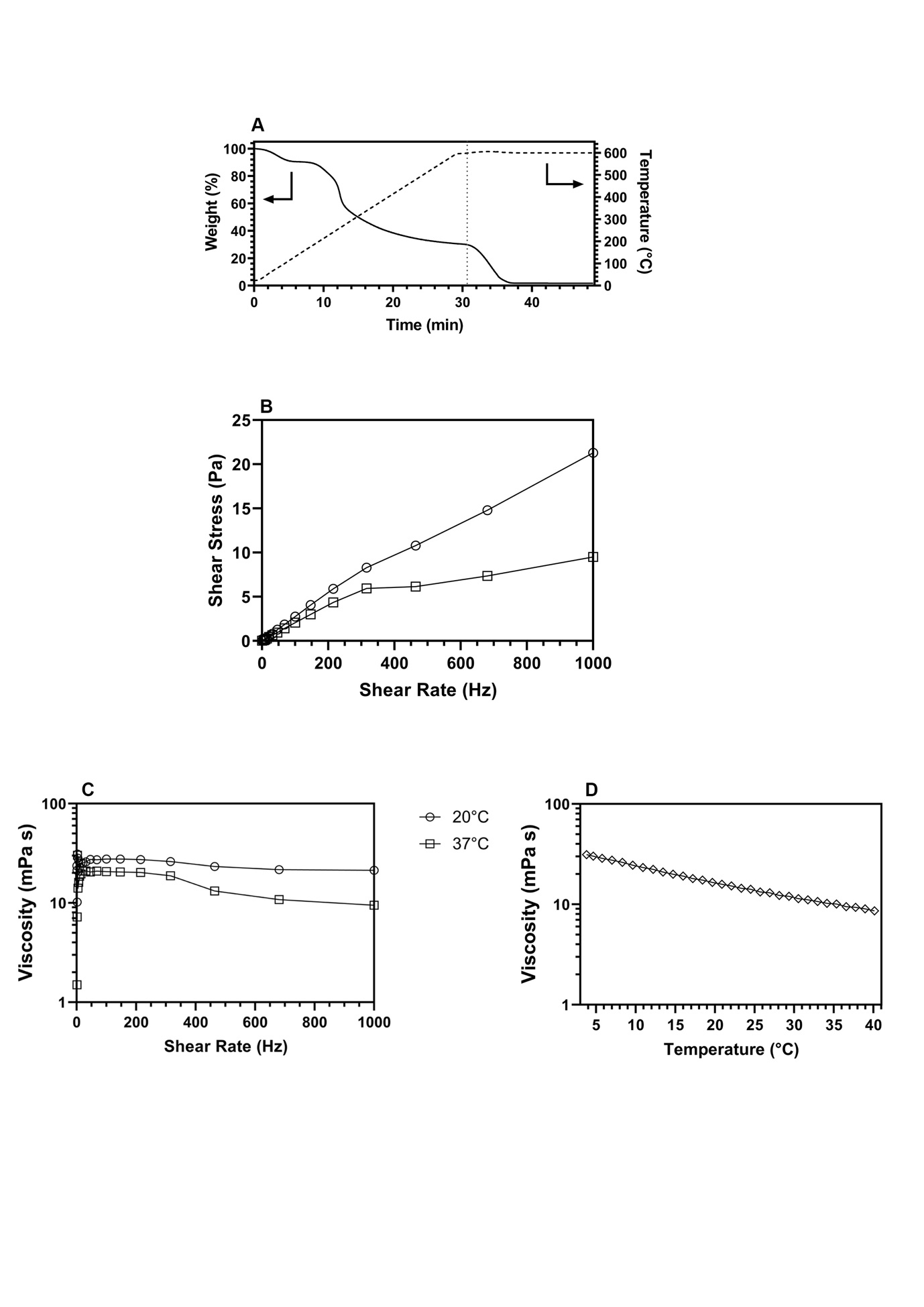
Figure S8. Characterization of GNR@Chit.**

**A**) Thermogram of GNRs@Chit decomposition. The dashed line corresponds to the switch from N_2_ atmosphere to air. The TGA profile shows a first loss corresponding to -10% weight loss due to the desorption of residual humidity from the lyophilization process. After keeping the sample at 600 °C for 15 min in air, the residual mass, corresponding to the total inorganic content, was as low as 2.1 wt. %.

**B**) Shear stress-shear rate and **C**) viscosity-shear rate rotational rheology analysis of GNRs@Chit at 20°C and 37°C. At low shear rates (< 300 Hz) the linearity of the shear stress – shear rate curve suggests Newtonian behaviour of the fluid, confirmed by the fact that viscosity is constant in that range. At higher shear rates viscosity decreases, revealing shear-thinning behaviour with higher applied stresses, and this phenomenon is even more evident at 37°C.

**D**) Dependence of viscosity with temperature, measured at a constant shear rate of 50 Hz.


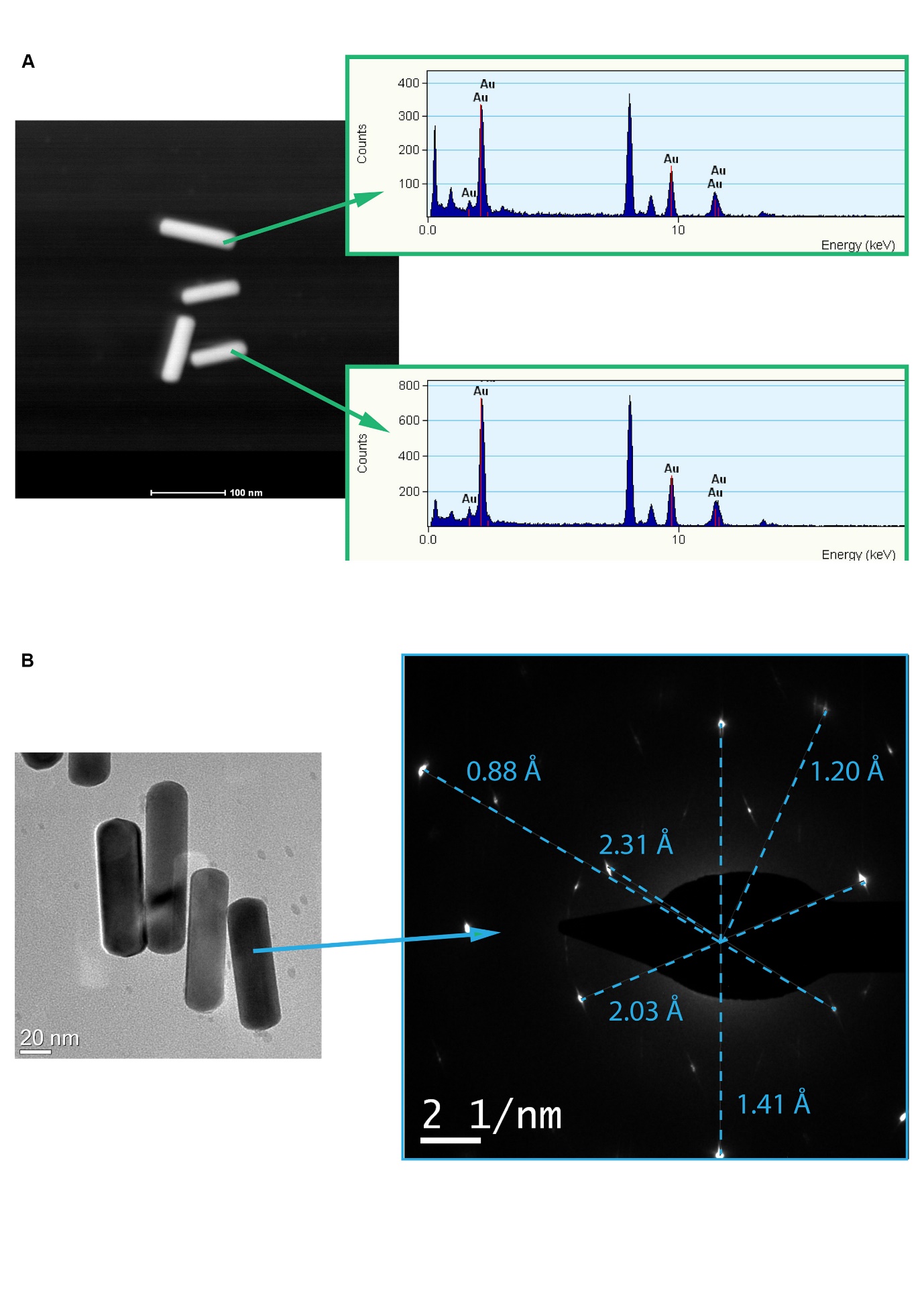


**Figure S9. TEM analysis of GNRs@Chit-*Iso4*.**

**A)** High Angle Annular Dark Field Detector (HAADF) image of GNRs@Chit-*Iso4* and the results of Energy-dispersive X-ray spectroscopy (EDX) analysis performed on two isolated GNRs, revealing gold as the only detected atomic component of the GNRs core, with no bromine coming from cytotoxic CTAB was detected. Unlabeled peaks are related to copper and carbon from the sample supporting TEM grid.

**B)** TEM image of GNRs@Chit-*Iso4* and the corresponding selected-area electron diffraction (SAED) pattern confirming the conservation of the crystallinity of the gold core. The reflections corresponding to 1.20 Å, 1.41 Å, 2.03 Å and 2.31 Å have been assigned to the [311], [220], [200] and [111] sets of planes, respectively, by comparing the interplanar distances with tabulated literature data [7].

**Figure S10. Representative** **HPLC chromatogram of the hydrolyzed products obtained from GNRs@Chit-Iso4 (A) and GNRs@Chit-Cys (B).**

Lyophilized GNRs (0.5-1 mM of Au, 0.5-1 x10^11^ NPs/ml) were suspended in water at 5 mg/ml (based on their dry-matter content) and an aliquot was subjected to acidic hydrolysis (20 hours at 110°C, in 6 M hydrochloric acid, 0.1% phenol, 0.1% thioglycolic acid under reduced pressure in an atmosphere nitrogen). The amino acid content in the hydrolyzed product was then quantified by ion exchange chromatography and post-column derivatization with ninhydrin. *Red arrows* indicate the amino acid type (*one letter code*) clearly detected and quantified over the background (compare with **panel B**). Amino acid in grey color (*three letter code*) corresponds to the elution time of a standard amino acid mixture used for calibrating the column. Amino acid in purple color (*three letter code*) are below the detection limit. Sarcosine (*Sar*) was added to the sample as an internal control standard. Note that for d-phg no reference standard exists and in this case the d-phg elutes as methionine; moreover, Cys cannot be quantified using this method.

**
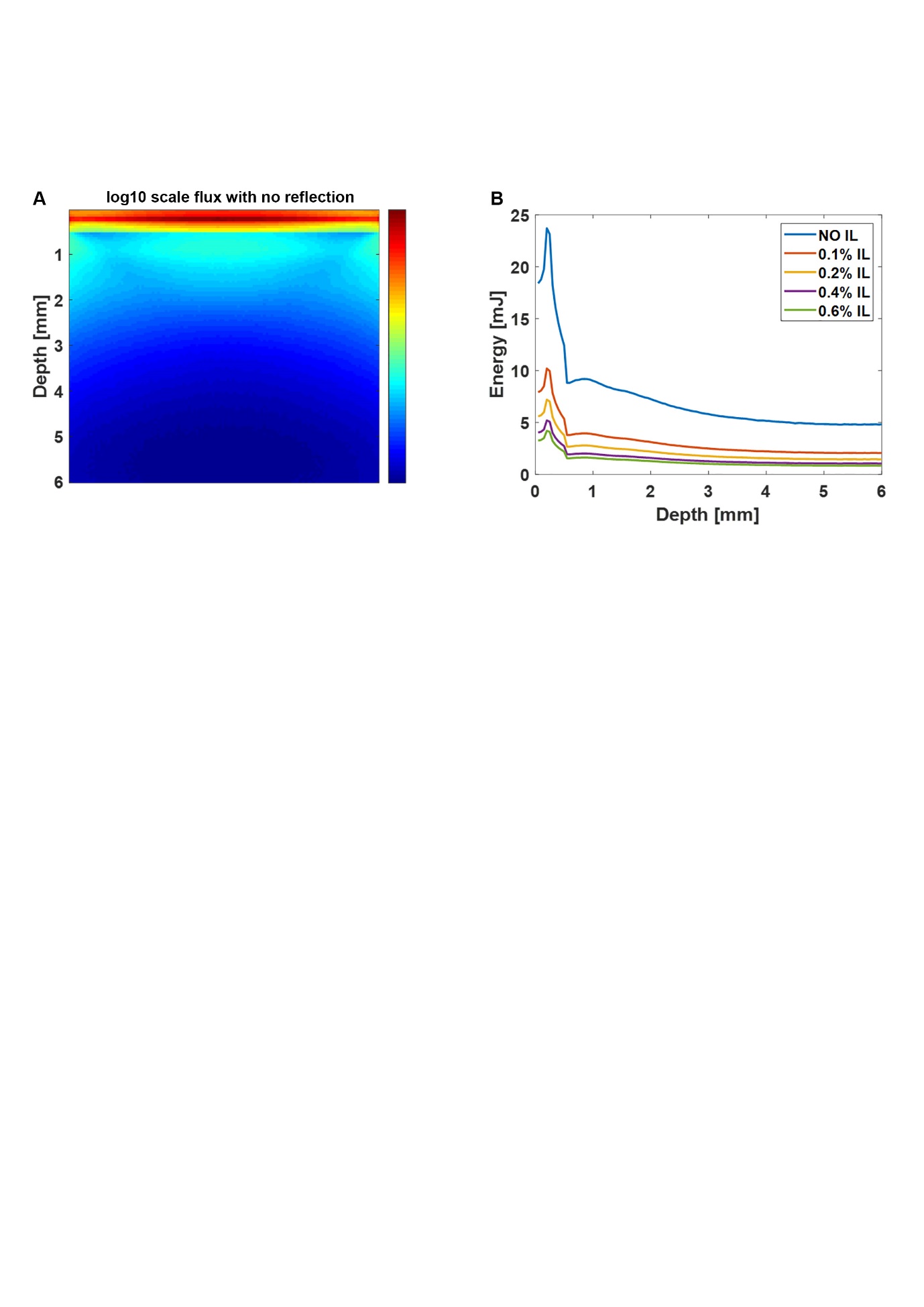
Figure S11. Monte Carlo model of light transport.**

**A**) Light fluence distribution within the simulated domain that consists of skin line and the standard tissues. The simulated image shows a very good distribution of light fluence where more fluence is observed at the skin line and then decays along the depth. The color bar shows the fluence distribution where red represents the high fluence and black represents very low fluence.

**B**) Averaged energy distribution along the depth considering the presence of different IL layers. The light is slightly collimated after penetrating the tissue interface; slightly deeper, both incident and backscattered light are contributing, causing a peak of fluence, which then decays exponentially.

**
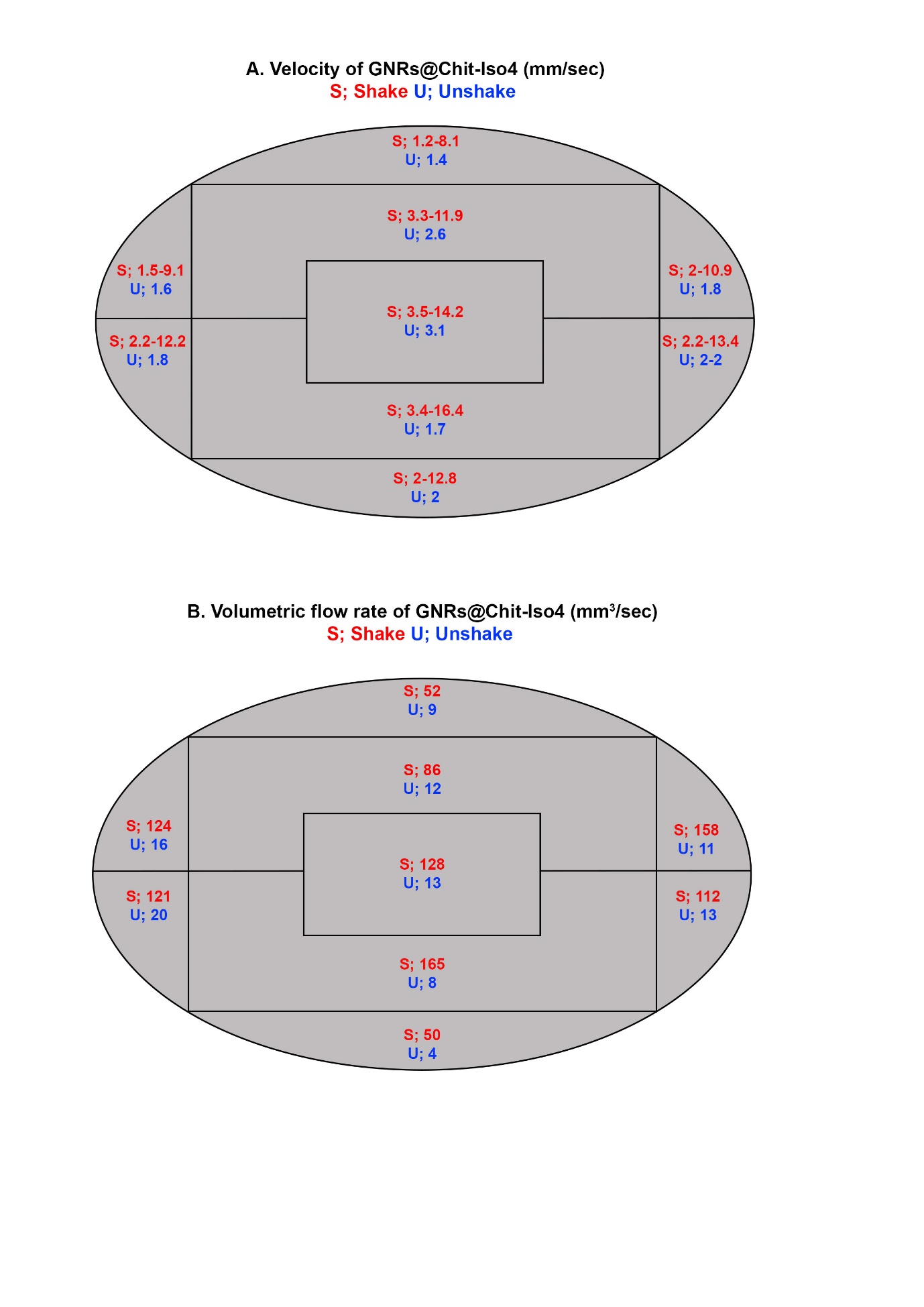
Figure S12. Velocity and volumetric flow rate of GNRs@Chit-*Iso4* in the absence and presence of US-assisted shaking. A**) velocity and **B**) volumetric flow rate were measured for 5 seconds during the 3 minutes of agitation, then analyzed in the total volume and in the different sectors of the bladder volume. Values are showing the mean from 4 animals each condition, with SEM <15%.

**
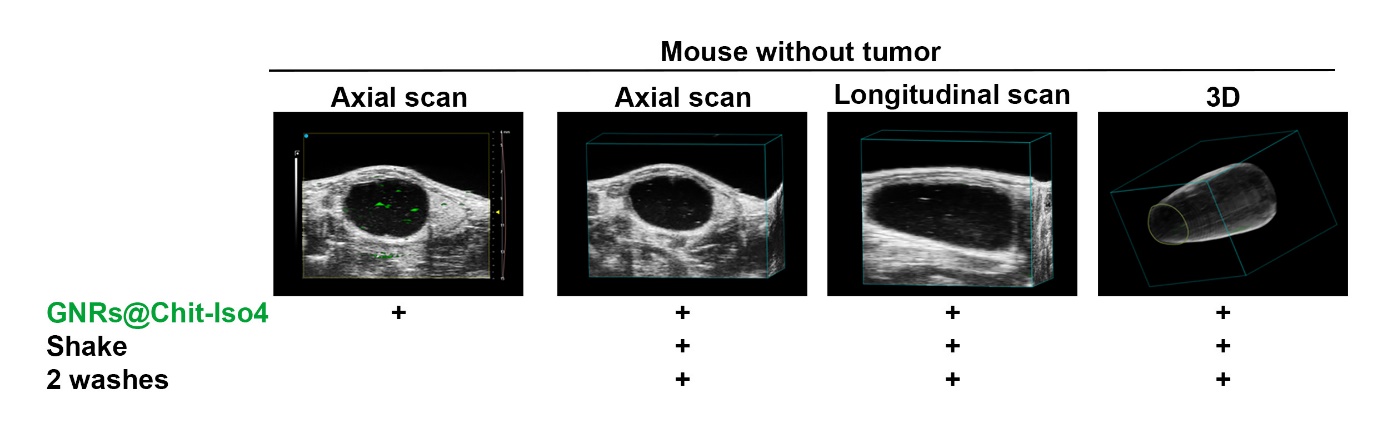
**

**Figure S13. GNRs@Chit-Iso4 does not bind to normal urothelium *in vivo*.**

PAI of GNRs@Chit-Iso4 (3 nmol Au) immediately after instillation, after instillation followed by low frequency US-assisted shaking and washes (axial and longitudinal scans and 3D PAI) in a healthy mouse bladder. One representative mouse out of four analyzed is shown.


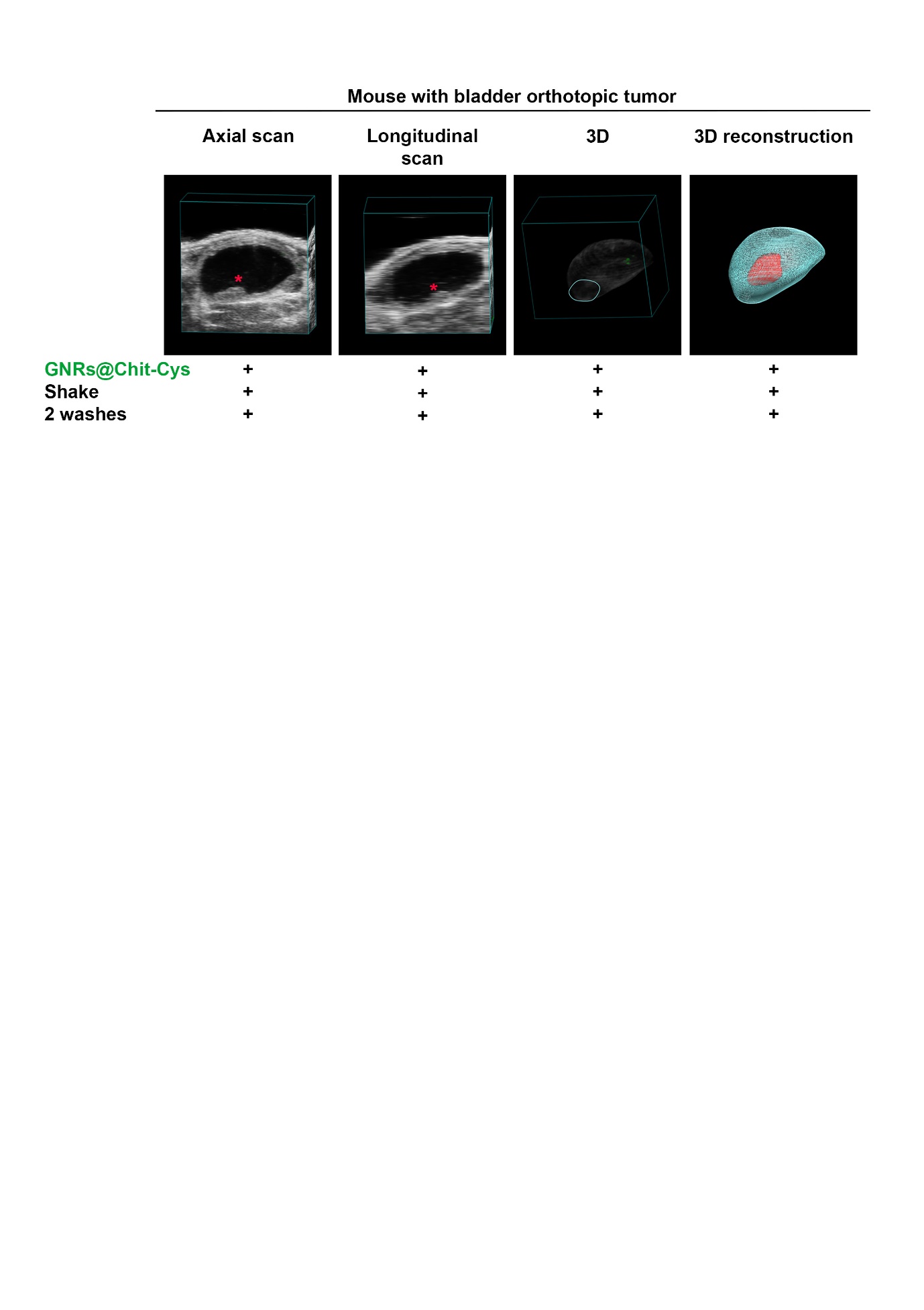


**Figure S14. GNRs@Chit does not bind to orthotopic MB49-Luc tumor.**

PAI of GNRs@Chit (3 nmol Au) after instillation followed by low frequency US-assisted shaking and washes (axial and longitudinal scans and 3D PAI) in a mouse bladder bearing MB49-Luc tumor (red asterisks). One representative mouse out of four analyzed is shown.

**
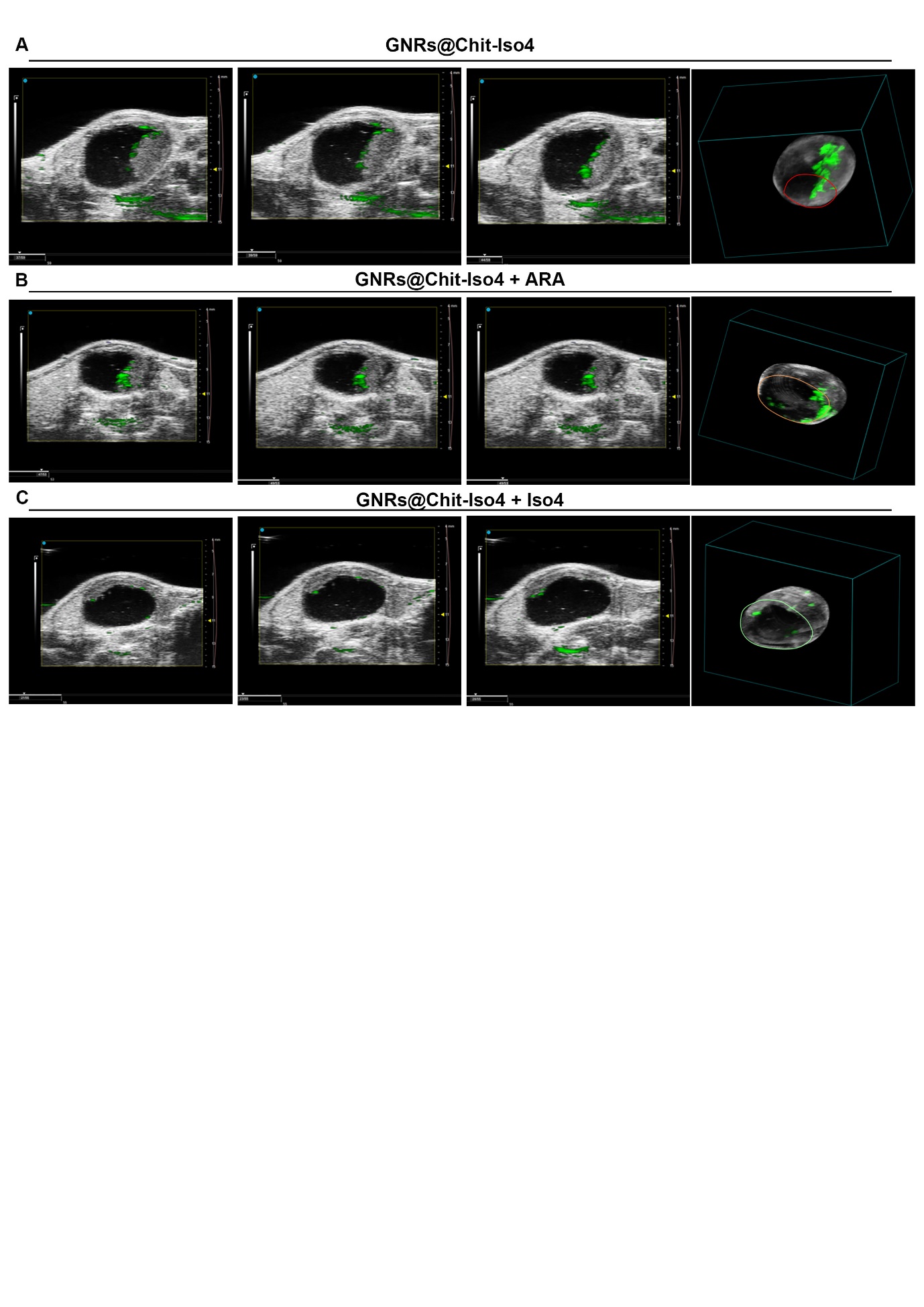
Figure S15. The binding of GNRs@Chit-Iso4 to neoplastic urothelium is specific.**

Three representative axial frames and 3D PAI of GNRs@Chit-Iso4 (1.5 nmol Au, containing 5.6 µg of Iso4) (**A**), added of 296 µg ARA peptide (**B**), or 296 µg Iso4 peptide (**C**) after intravesical instillation followed by US-assisted shaking and two washes. One representative mouse of two is shown for each condition, and animals with comparable bioluminescence were used (total flux [p/s] of 7.2x10^7^ in panel **A**, 1.22x10^7^ in panel **B**, and 2x10^7^ in panel **C**).

**Supplementary Videos**

**

- **Video SV1. Settling of GNRs in the murine bladder during US imaging.** The urinary content is mainly concentrated in the lower half of the bladder or accumulated at the bottom of the bladder. The ultrasound probe is placed on the abdomen of the animal.

- **Video SV2. US-assisted shaking improves the distribution of GNRs in the bladder.** The urinary content is more distributed in the urinary lumen during the US-assisted shaking. The probe for US-assisted shaking and the probe for US imaging are placed on the abdomen of the animal at 45 degrees from each other as reported in Supplementary Figure S2.

**Supplementary references**

[1] X. Ye, C. Zheng, J. Chen, Y. Gao, C.B. Murray, Using binary surfactant mixtures to simultaneously improve the dimensional tunability and monodispersity in the seeded growth of gold nanorods, Nano Lett 13(2) (2013) 765-71.

[2] M.E. Gallina, Y. Zhou, C.J. Johnson, D. Harris-Birtill, M. Singh, H. Zhao, D. Ma, T. Cass, D.S. Elson, Aptamer-conjugated, fluorescent gold nanorods as potential cancer theradiagnostic agents, Mater Sci Eng C Mater Biol Appl 59 (2016) 324-332.

[3] P. Manivasagan, S. Bharathiraja, N.Q. Bui, I.G. Lim, J. Oh, Paclitaxel-loaded chitosan oligosaccharide-stabilized gold nanoparticles as novel agents for drug delivery and photoacoustic imaging of cancer cells, Int J Pharm 511(1) (2016) 367-379.

[4] J. Sharifi-Rad, C. Quispe, M. Butnariu, L.S. Rotariu, O. Sytar, S. Sestito, S. Rapposelli, M. Akram, M. Iqbal, A. Krishna, N.V.A. Kumar, S.S. Braga, S.M. Cardoso, K. Jafernik, H. Ekiert, N. Cruz-Martins, A. Szopa, M. Villagran, L. Mardones, M. Martorell, A.O. Docea, D. Calina, Chitosan nanoparticles as a promising tool in nanomedicine with particular emphasis on oncological treatment, Cancer Cell Int 21(1) (2021) 318.

[5] E.H. Ooi, V. Popov, M. Alfano, J.K.K. Cheong, Influence of natural convection on gold nanorods-assisted photothermal treatment of bladder cancer in mice, Int J Hyperthermia 37(1) (2020) 634-650.

[6] Z.X. M.Lavertu, A.N.Serreqi, M.Berrada, A.Rodrigues, D.Wang, M.D.Buschmann, AjayGupta, A validated 1H NMR method for the determination of the degree of deacetylation of chitosan, 32(6) (2003) 9.

[7] H.O. In-Kook Suh, Y. Waseda High-temperature thermal expansion of six metallic elements measured by dilatation method and X-ray diffraction, Journal of Materials Science volume 23 (1988) 3.
